# Computational Study of Amyloidβ_42_ Familial Mutations and Metal Interaction: Impact on Monomers and Aggregates Dynamical Behaviors

**DOI:** 10.1021/acs.inorgchem.3c04555

**Published:** 2024-02-26

**Authors:** Lorena Roldán-Martín, Mariona Sodupe, Jean-Didier Maréchal

**Affiliations:** Departament de Química, Universitat Autònoma de Barcelona, 08193 Cerdanyola del Vallès, Spain

## Abstract

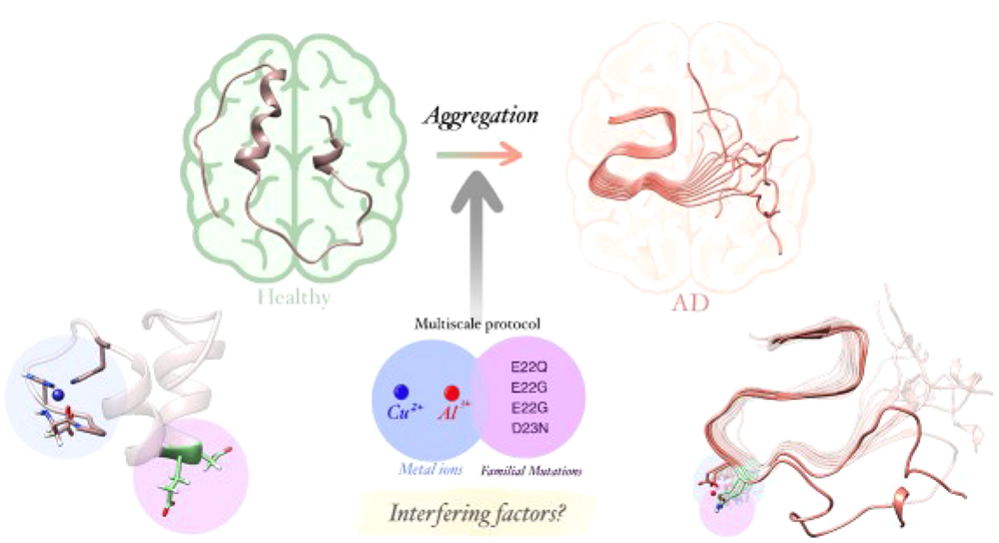

One of the main hallmarks of Alzheimer’s Disease is the formation of β-amyloid plaques, whose formation may be enhanced by metal binding or the appearance of familial mutations. In the present study, the simultaneous effect of familial mutations (E22Q, E22G, E22K, and D23N) and binding to metal ions (Cu(II) or Al(III)) is studied at the Aβ_42_ monomeric and fibrillar levels. With the application of GaMD and MD simulations, it is observed that the effects of metal binding and mutations differ in the monomeric and fibrillar forms. In the monomeric structures, without metal binding, all mutations reduce the amount of α-helix and increase, in some cases, the β-sheet content. In the presence of Cu(II) and Al(III) metal ions, the peptide becomes less flexible, and the β-sheet content decreases in favor of forming α-helix motifs that stabilize the system through interhelical contacts. Regarding the fibrillar structures, mutations decrease the opening of the fiber in the vertical axis, thereby stabilizing the S-shaped structure of the fiber. This effect is, in general, enhanced upon metal binding. These results may explain the different Aβ_42_ aggregation patterns observed in familial mutations.

## Abstract 2

The amyloid-β aggregation process is still widely unknown and difficult to experimentally determine. This work uses a multiscale computational protocol to study possible interfering factors on the aggregation process. The focus is placed on the effect of Cu and Al metal ions, together with four well-known familial mutations (E22Q, E22G, E22K, and D23N), on the monomeric and fibrillar species.

## Introduction

Nowadays, Alzheimer’s Disease (AD) is the most common form of dementia, and its incidence is expected to continue growing in the following years.^1^ One of the main hallmarks of its diagnosis is the detection of extracellular plaques formed by the β-amyloid peptide (Aβ). Aβ fragments are produced by the proteolytic cleavage of the Amyloid Precursor Protein (APP) by β- and γ-secretases, producing Aβ fragments that range from 39 to 42 residues long. Most AD cases start from unknown reasons, but 1% corresponds to familial cases with well-characterized mutations. Such mutations can be found in the preprocessing step, in the cleavage of APP, or in the peptide itself. From a molecular perspective, four familial Aβ mutations located in residues 22 and 23 are of particular interest as they are suspected to impact the peptide’s secondary structure and modify its aggregation pattern.^2,3^

In most populations, position 22 is occupied by glutamic acid. Patients with Arctic mutations (E22G) display an enhanced formation of small Aβ protofibrils and oligomers but a lower aggregation rate. In contrast, those with the Dutch mutation (E22Q) present the formation of amorphous fibrils and oligomers with a faster aggregation rate, even faster than E22. The Italian mutation (E22K) presents an accelerated aggregation rate to oligomers but with fewer fibrils.^2,4^ Position 23 is occupied by aspartic acid, whose Iowa mutation (D23N) results in a higher aggregation rate than the WT, with a characteristic bend motif that could affect folding and aggregation.^5^ It has been suggested that with mutation D23N, the electrostatic repulsion between monomers will be reduced since the overall charge of the peptide is minor, increasing the self-aggregation rate.^6^ In fact, all of these mutations reduce the negative charge of the peptide. Unveiling the exact mechanism by which each mutation modifies the aggregation pattern is highly interesting, because it may lead to a better understanding of AD development.

How the aggregation process starts and propagates is still an open question with several lines of focus. The so-called amyloid cascade and metal-ion hypotheses are the more debated ones. The amyloid cascade states that the Aβ imbalance is the central event that initiates the pathology and is associated with an inflammatory response and increased oxidative stress.^7^ The metal-ion hypothesis sustains the crucial role of metal-Aβ complexes in stabilizing amyloid plaques, and most stand on much evidence collected over the years of high concentrations of metal ions in postmortem brain tissues.^8^

Several metals are shown to play a role in the process of Aβ aggregation. Some are part of the biological panoply of life; others are environmental and industrial contaminants.^9^ Copper and iron can be classified in the first family, while aluminum and cadmium are part of the second.^10^ Experimental and theoretical works have been intensively performed to ascertain Metal-Aβ interactions.^11−18^ Among relevant observations is how aggregates are sensitive to the type of metal and their concentrations in the medium. Remarkably, many efforts have been devoted to finding curative drugs for AD, focusing on those Metal-Aβ interactions, with infructuous results until now. Some of the most recent trials are metal chelators, small bifunctional molecules targeting metals, and Aβ and fibrillar structure disruption.^19,20^ However, it is considered that metal homeostasis is a highly complicated biological process that cannot be tackled as a simple excess of metals addressed with chelators.^21^ Therefore, an in-depth study of the aggregation process, considering its structure and the factors by which it is governed, can be critical for future treatments. Variations in the aggregation process are significant. After years of experimental and theoretical investigations, progress has been made in decoding the main tendencies in the first coordination sphere of each metal, especially in monomeric species,^22,23^ but at which stage and to what degree they impact aggregation processes is still under debate.

Computational tools are opening avenues at the interface between chemistry and biology. Regarding peptide systems such as Aβ, their main challenges arise from their high conformational flexibility, which complicates identifying even simple geometric patterns. Some groups have tried to get insights with reasonable success.^24−28^ In our previous study, the effect of Cu(II) and Al(III) binding on the WT monomeric form of Aβ_42_ was already described, with certain differences depending on the metal cation; i.e., metal binding remarkably reduces the flexibility of the peptide, with Cu(II) coordination increasing the α-helix content while Al(III) reducing it.^29^ Besides, we have also reported the effect of Cu(II) and Al(III) binding on the fibrillar form of the Aβ_42_, demonstrating that Al(III) binding has a stabilizing effect. At the same time, Cu(II) coordination partially disrupts the fiber structure.^30^ Concerning the familial mutations, the changes in the secondary structure of the peptides derived from the E22 mutations have already been explored, though mostly centered on the comparison between the mutated Aβ_42_ and Aβ_40_ forms.^31^ The dimerization of the E22Q mutated form has also been computationally explored, though only in the 16–22 fragment.^32^ Some experimental studies also report the effect of the mutations in Aβ40 fibrillar forms.^33,34^ However, to the best of our knowledge, no studies on the influence of mutations on the complete fibrillar form and the interrelation between mutations and metal binding, two of the main accepted aggregation hypotheses, have been reported.

Here, we analyze the monomeric and fibrillar structures of Aβ_42_ with aggregation-prone familial mutations E22Q, E22G, E22K, and D23N, simultaneously bound to Cu(II) or Al(III) binding. For that, an extensive conformational analysis is performed by means of Gaussian accelerated Molecular Dynamics (GaMD) and classical Molecular Dynamics simulations (MD). This work is expected to shed light on the impact of specific interactions in monomeric and multimeric species to understand the role of these mutations better.

## Results and Discussion

The familial mutations in this study are clustered at positions 22 and 23, with the variants E22G, E22Q, E22K, and D23N. All have been reported to highly impact the aggregation profile of the peptide.^2,5^ Although our work aims at providing molecular insights throughout computation, simulations of the entire metal-dependent aggregation process that consider the total flexibility of the monomers, as well as their assembly to form metal-containing fibrillar or amorphous structures, are still a far-reaching challenge of molecular modeling. However, addressing separate monomeric species, on one side, and fibrillar structures, on the other, can yet lead to relevant insights and has been the working hypothesis of this study. That is, the impact of the mutations on monomeric and aggregate structures and their relationship with metal-binding have been studied. For monomeric species, we expect that the mutations would primarily affect the secondary structure. Those effects could further alter the subsequent aggregation process. For aggregates, the mutations were expected to affect both the stability of the tertiary structure and the metal-binding patterns. For the sake of this article, monomeric species and aggregates are presented in individual blocks.

### Monomeric Species

**Figure 1:**
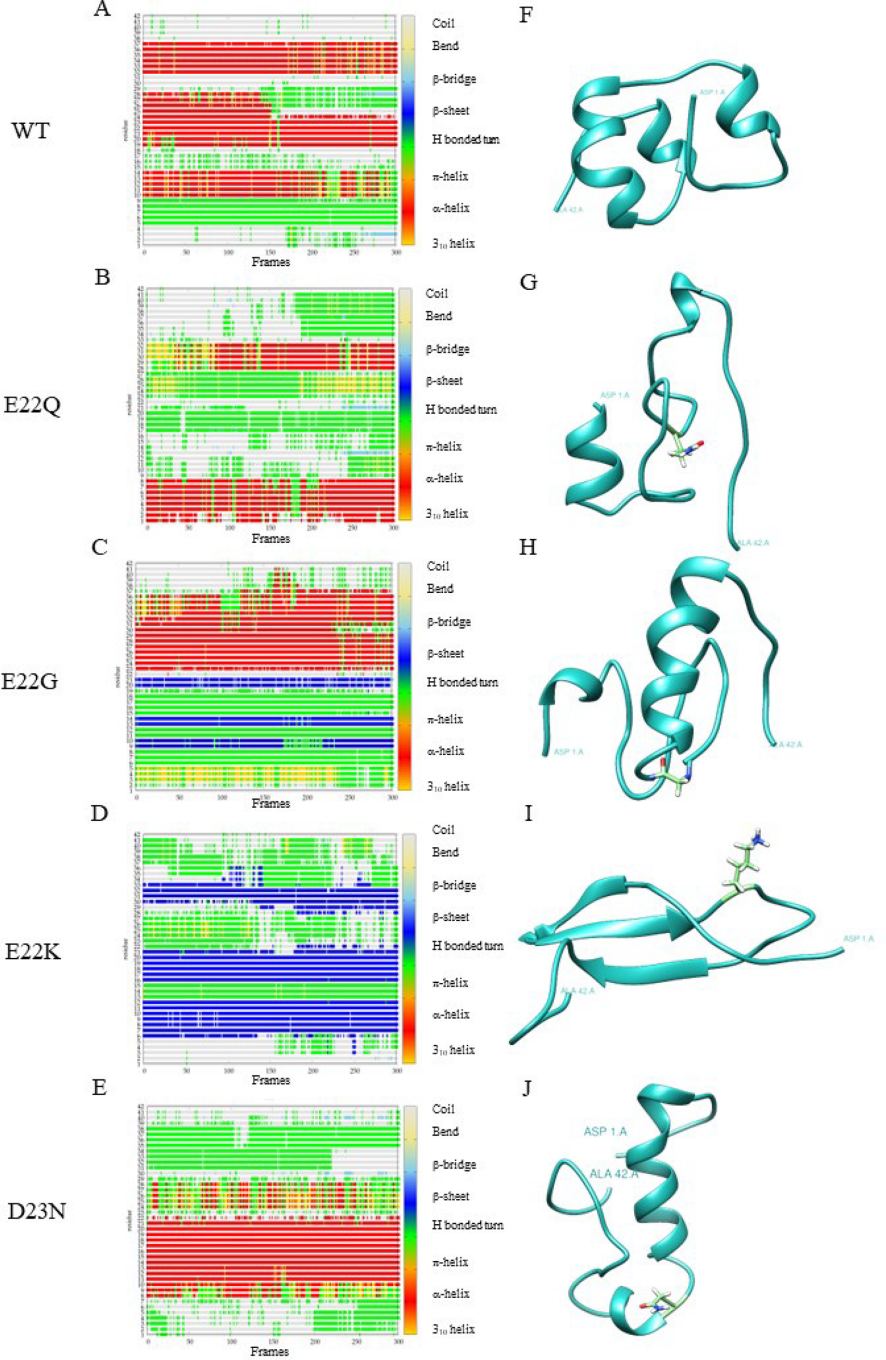
A-E Timeline analysis of the MD trajectory for FP WT, E22Q, E22G, E22K, and D23N mutations. The β-sheets correspond to blue regions. F-J Representative structures of the most populated cluster from the MD of each variant. The mutated residue, when present, is colored light green.

**Figure 2:**
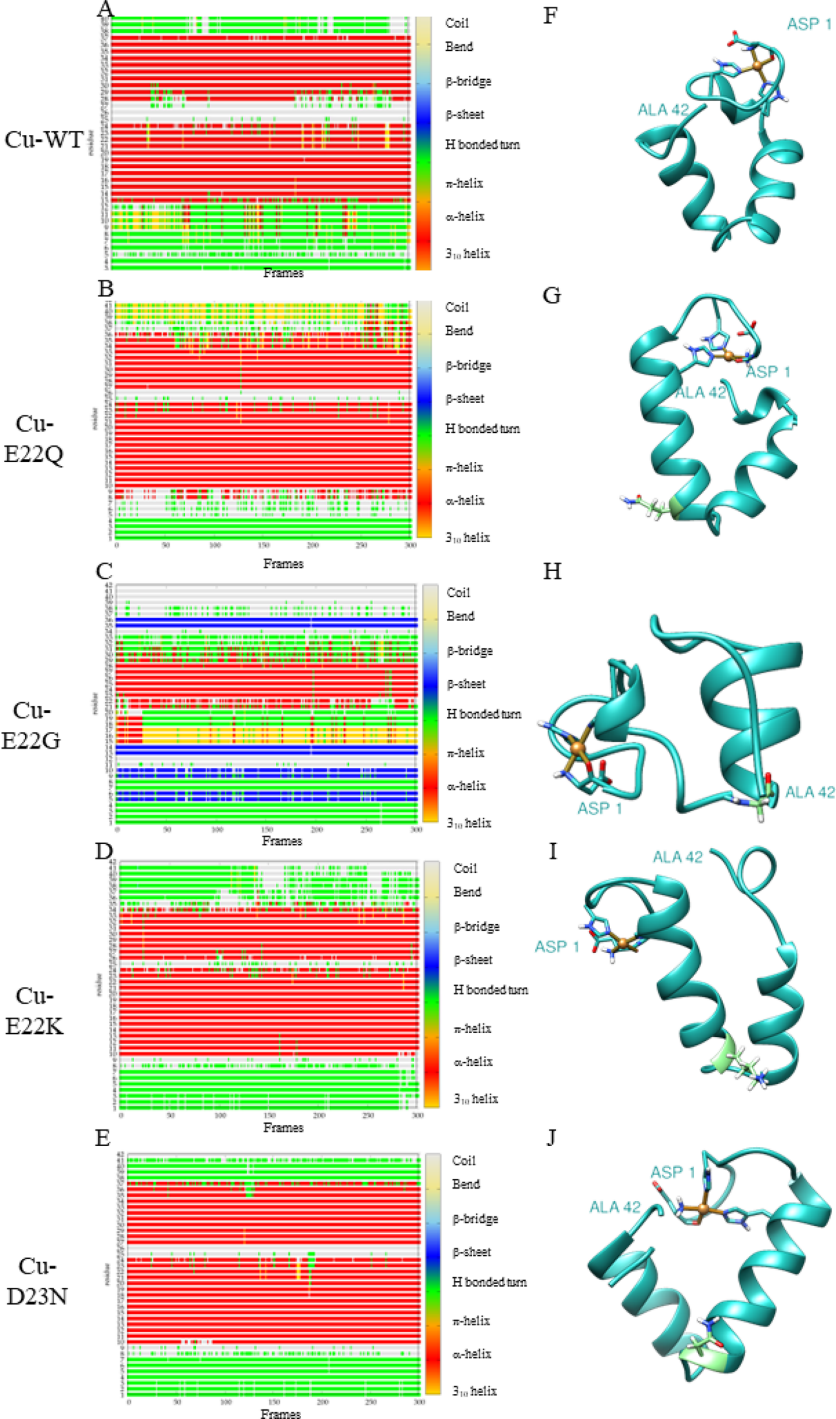
A-E Timeline analysis of the MD trajectory for Cu-WT, Cu-E22Q, Cu-E22G, Cu-E22K, and Cu-D23N mutations. The β-sheets correspond to the blue regions. F-J Representative structure of the most populated cluster from the MD of each variant. The mutated residue, when present, is represented in light green.

**Figure 3:**
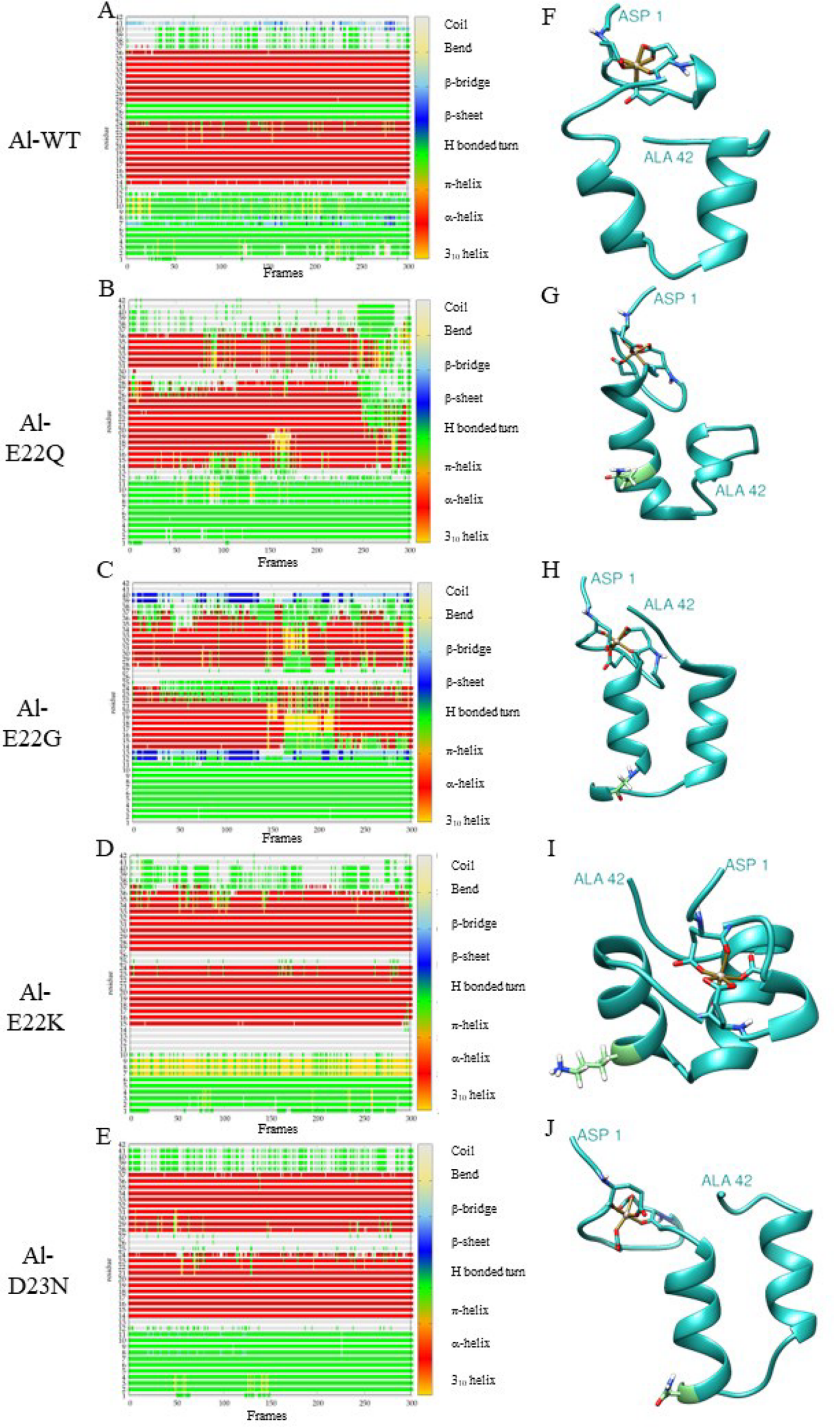
A-E Timeline analysis of MD trajectory for Al-WT, Al-E22Q, Al-E22G, Al-E22K, and Al-D23N mutations. The β-sheets correspond to the blue regions. F-J Representative structure of the most populated cluster from the MD of each variant. The mutated residue, when present, is represented in light green.

Structural descriptors for the monomeric species of the metal-free peptide, Cu(II), and Al(III) systems are summarized in Tables 1, 2, and 3, respectively. These descriptors are i) the α-helix and ii) β-sheet contents obtained along the trajectories, which inform about the secondary structure adopted by the peptides, iii) the U-shaped percentage, related to the tertiary structure of the monomers and linked to higher aggregation structures,^35^ iv) the Hydrogen Bond (HB) contacts, primarily situated in the turn (Glu22-Lys28) of the U-shape, since they have been reported to be essential for the nucleation of Aβ,^36^ and v) the radius of gyration, related to the flexibility of the peptide. Such parameters are expected to be modified upon introduction of the familial mutations in the Aβ sequence. Results for the WT Aβ_42_ peptide^29^ have also been included for comparison. Figures 1, 2, and 3 show the timeline analysis and the representative structure of the most populated cluster of the metal-free peptide and the Cu(II) and Al(III) bound systems, respectively. Stability analysis including energy profile, contact map, RMSD to all, principal component analysis (PCA), and cluster countering for each complex is provided in the SI (Figures S1-S15).

**Table 1:** Results for the Metal-Free Peptide Monomeric System

System	α-hel.^a^	β-sheet^b^	HB (16–42)^c^	U-shape (%)^d^	RoG (Å)^e^
FP WT	48.8%	0%	**None**	0%	23.1 ± 10.7
FP E22Q	12.8%	0%	**Asp23-Ser26** (af 47.0%)	0%	23.0 ± 8.2
FP E22G	48.2%	6.8%	**None**	0%	23.5 ± 8.5
FP E22K	0%	38.9%	**Asp23-Ser26** (af 19.7%)	0%	25.7 ± 9.2
FP D23N	30.9%	0%	**Asn23-Ser26** (af 91.8%)	0%	25.6 ± 9.8

a α-Helix.

b β-Sheet percentage along the trajectory.

c HB contacts with an average frequency of at least 10%, suggested to participate in the U-shaped maintenance.

d Percentage of the U-shaped structure along the trajectory, counted by adding up the percentage of clusters with such a structure.

e Average Radius of Gyration along the MD trajectory and its standard deviation.

**Table 2:** Results for the Cu(II)-Bound Monomeric System

System	α-hel.^a^	β-sheet^b^	HB (16–42)^c^	U-shape (%)^d^	RoG (Å)^e^
Cu-WT	69.1%	0%	**Glu22-Lys28** (af 23.7%)	100%	15.0 ± 0.7
Cu-E22Q	69.2%	0%	**None**	100%	18.33 ± 1.8
Cu-E22G	33.8%	7.7%	**None**	0%	17.53 ± 1.0
Cu-E22K	66.3%	0%	**None**	100%	18.3 ± 3.0
Cu-D23N	74.5%	0%	**None**	100%	16.7 ± 1.7

a α-Helix.

b β-Sheet percentage along the trajectory.

c HB contacts with an average frequency of at least 10%, suggested to participate in the U-shaped maintenance.

d Percentage of the U-shaped structure along the trajectory, counted by adding up the percentage of clusters with such a structure.

e Average Radius of Gyration along the MD trajectory and its standard deviation.

**Table 3:** Results for the Al(III)-Bound Monomeric System

System	α-hel.^a^	β-sheet^b^	HB (16–42)^c^	U-shape (%)^d^	RoG (Å)^e^
Al-WT	68.3%	0.3%	**None**	96.7%	18.9 ± 1.8
Al-E22Q	55.2%	0%	**None**	71.4%	22.6 ± 5.1
Al-E22G	51.9%	2.4%	**None**	76.3%	19.6 ± 1.7
Al-E22K	71.0%	0%	**None**	100%	17.6 ± 1.8
Al-D23N	70.5%	0%	**Glu22 – Lys28** (af 73.7%)	99.7%	20.3 ± 1.5

a α-Helix.

b β-Sheet percentage along the trajectory.

c HB contacts with an average frequency of at least 10%, suggested to participate in the U-shaped maintenance.

d Percentage of the U-shaped structure along the trajectory, counted by adding up the percentage of clusters with such a structure.

e Average Radius of Gyration along the MD trajectory and its standard deviation.

For the metal-free peptide structures, all four mutations either dramatically reduce (E22Q, E22K, and D23N) or maintain (E22G) the α-helix content (Table 1 and Figure 1). The β-sheet content is increased in mutations E22G and E22K. This is particularly striking for E22K as the β-sheet content rises to 38.9% with three consistent regions (residues 6–12, 17–21, and 30–33) (Figure 1), which induces the disappearance of the α-helix content (0%). However, the mutated systems’ flexibility is similar to the WT’s, according to the similar Radius of Gyration (23.1 Å for the WT and between 23.0 and 25.7 Å for the variants) and the significant standard deviation. Furthermore, hydrogen bonds (HBs) in the U-turn, known for their role in promoting aggregation,^36^ appear with all mutations except E22G (47.0% in E22Q, 19.7% in E22K, 91.8% in D23N), while in the WT they were not formed. The appearance of HBs in such systems does not correlate with the percentage of the U shape, as initially expected. Thus, the data show that mutations have a clear impact on the general fold of the metal-free peptide, which could already suggest significant changes in how the system preorganizes for metal-binding and/or aggregation processes.

Upon metal coordination, the effects observed correspond to those already reported in our previous work:^29^ both Cu(II) and Al(III) coordinations reduce the Radius of Gyration, producing less flexible structures, and increase their α-helix content. Indeed, metal binding redistributes the helical regions, leading to U-shaped structures that are stabilized by hydrophobic interhelical contacts. Here, comparisons are performed among the complexes with the same coordinated metal ion to describe the effect of the mutations.

Cu-WT (Table 2, Figure 2) exhibits a high α-helix content (69.1%), involving residues from 13 to 24 and 28 to 37 and a 100% U-percentage. Regarding Cu-E22Q, Cu-E22K, and Cu-D23N mutated complexes, minor differences are observed in comparison to the Cu-WT complex, obtaining a similar α-helix content (69.2%, 66.3%, and 74.5%) and a 100% percentage of the U-shaped structure. On the contrary, Cu-E22G reduces the α-helix content and abolishes the U-shaped structure. Besides, only Cu-E22G produces several long-lasting β-sheet regions (7.7%), involving residues 5–6, 9–10, 13–14, and 35–36 (Figure 2) in agreement with glycine being a helix-breaker. It is remarkable that the Cu-E22K system does not retrieve any β-sheet content at all, while such a mutation caused a substantial increase in the metal-free system.

For Al(III) systems (Table 3, Figure 3), the Al-WT form shows both high α-helix content (68.3%) and U-shaped percentage (96.7%), with transitory β-sheet regions (0.3%) appearing along residues 7–8 and 40–41. Al-E22Q and Al-E22G show both a smaller α-helix (55.2% and 51.9%, respectively) and U-shaped content (71.4% and 76.3%) compared to Al-WT. For the Al-E22G variant, there is also a slight increase of the β-sheet content (2.4%), as two stable β-sheet regions are identified at residues 12–13 and 39–40. Both Al-E22K and Al-D23N produce a slight increase in the α-helix content (71.0% and 70.5%) and in the U-shape (100% and 99.7%), abolishing the β-sheet content (Figure 3D). In general, the timeline analysis (Figure 3) indicates that all Al-bound systems exhibit a less organized secondary structure pattern than the copper-containing systems, in agreement with the larger Radius of Giration values obtained for the formers. Such differences may be linked to Al(III) coordination introducing a higher charge than Cu(II), further affecting distal areas.

Overall, mutations at residues E22 and D23 and metal binding have different impacts on the structure and dynamics of the Aβ_42_ peptide. Without metal, all mutations reduce the amount of α-helix content and increase, in some cases, the β-sheet content. This may correlate with experimental observations showing faster aggregation for E22 mutants.^2^ The most striking case is the E22K variant for which stable β-sheet motifs (up to 39%) are observed during the trajectory. In the presence of Cu(II) and Al(III) metal ions, the peptide becomes less flexible, and the β-sheet content decreases in favor of forming α-helix motifs. Such helices are displaced toward the C_Ter_ end compared to the metal-free system. Once formed, such helices are stabilized by interhelical hydrophobic patterns, promoting the formation of U-shaped configurations. The only exception for both metals is Cu-E22G, for which the α-helix content decreases, while the β-sheet one increases. However, the differences between variants are not so evident in the presence of the metallic ions, with the effect of metal coordination being more important than the mutation itself. In fact, the stable β-sheet motifs observed in FP-E22K are not retrieved in the metallic systems.

### Fibrillar Species

**Figure 4:**
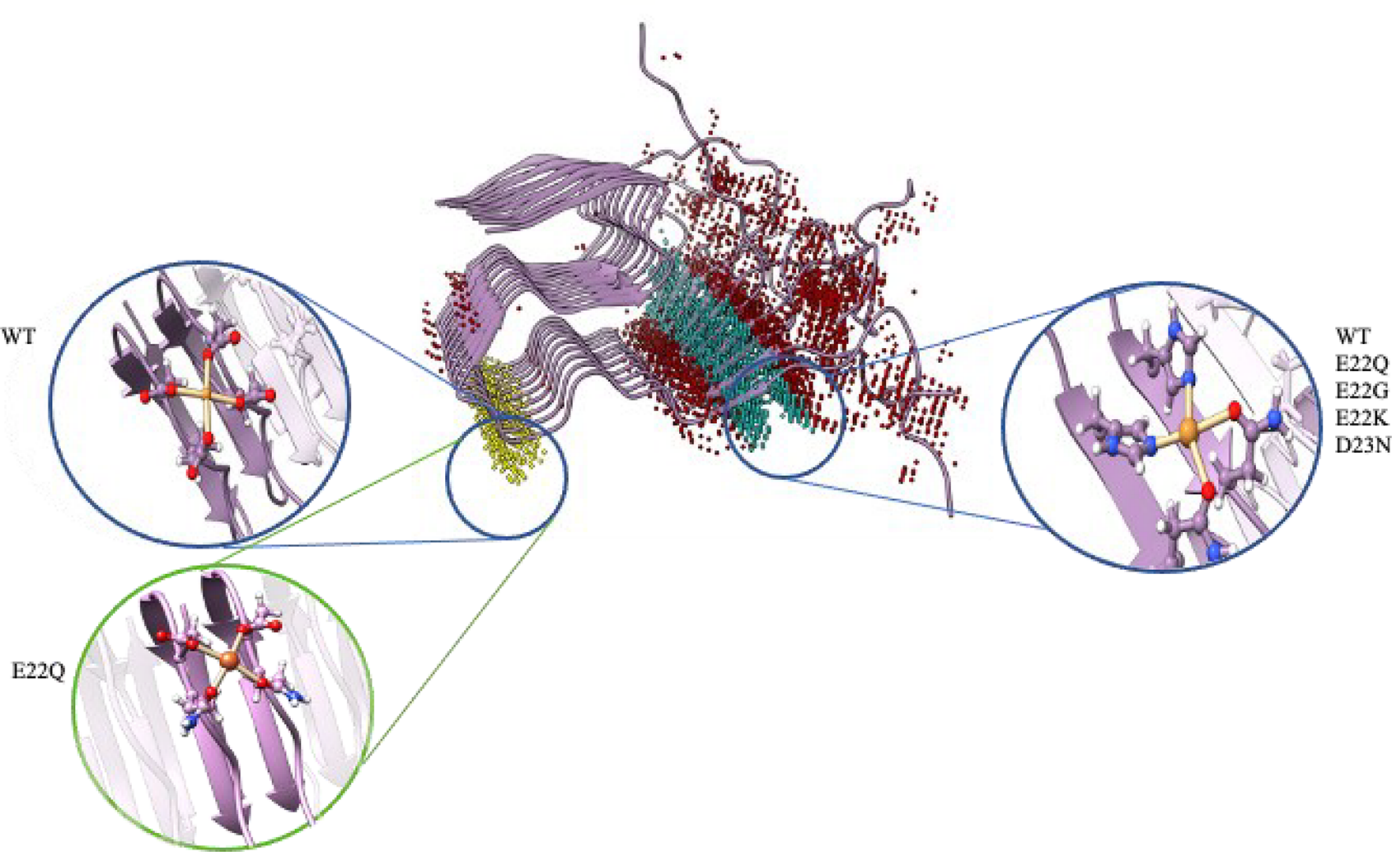
Representation of BioMetAll results. On the left, coordinations obtained for Al(III) bound to WT and E22Q variants are represented. On the right, coordination is found for all the Cu(II) bound complexes.

Our study’s next step aims to assess familial mutations’ and metal binding impact on fibrillar species. Since no crystallographic structures for the mutated Aβ fibrillar forms are available, the WT crystallographic structure has been selected as a starting conformation. For the metal-free and the Cu(II)-bound systems, it has been possible to study the four variants E22Q, E22G, E22K and D23N, maintaining the coordination sphere defined in our previous work (Figure 4).^30^ For the E22K, E22G, and D23N mutations, BioMetAll, a metal binding site predictor, was not able to find a compatible configuration for Al(III) binding since the mutated E22 and D23 residues, previously found in the metal coordination sphere, are substituted by Gly, Lys, or Asn, which do not allow a proper coordination.^30^ For E22Q, however, a feasible binding site that includes the mutated Q22 and D23 residues was predicted by BioMetAll (Figure 4). Hence, simulations with Al(III) bound to the fibrillar system were only possible for the E22Q variant.

**Figure 5:**
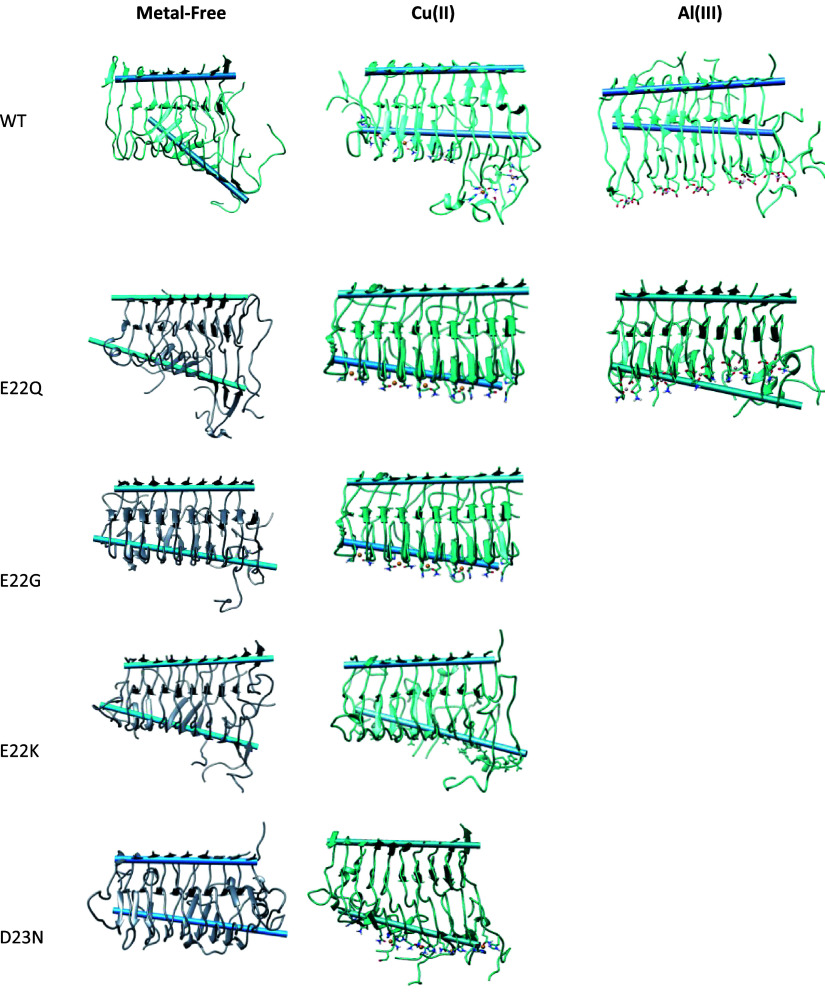
Most populated cluster obtained from the GaMD simulation for the metal-free, Cu(II), and Al(III) bound forms in WT, E22Q, E22G, E22K, and D23N variants. In blue, the two axes comprised of residues 39–41 (top) and residues 13–15 in β-sheets (bottom) are represented.

In our previous study, for all metal-free and metal-bound Aβ_42_ aggregates, a collective motion in the form of a fan-like displacement was identified.^30^ Present simulations for the four familial variants in metal-free forms show that the fan-like movement is maintained. Trajectory analyses are given in the SI (Figures S16-S19, S21-S24, S26). However, the calculation shows a lower amplitude, particularly for the E22G mutant (Figure 5) thereby indicating that the fibril is more rigid and less prone to disaggregate.

**Figure 6:**
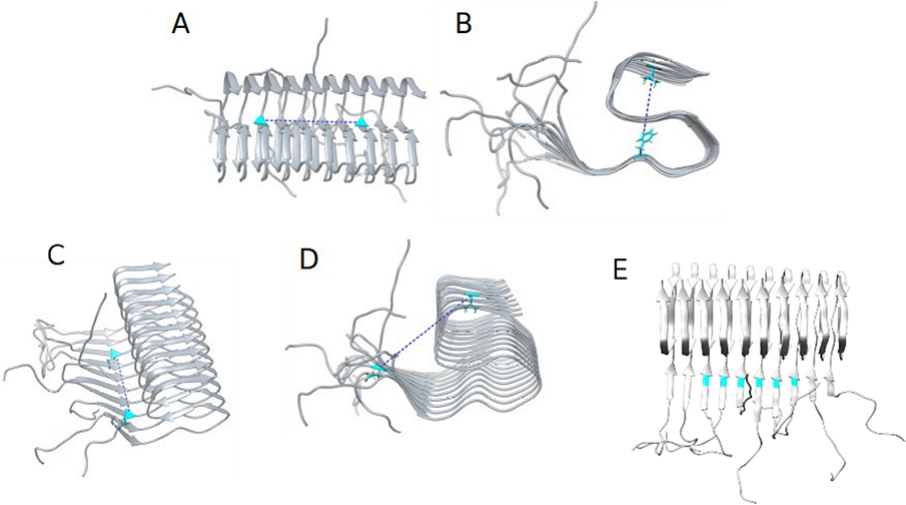
Illustrative cases for the five-distances measurement, in blue, represent the relevant residues for each measure. A. Distance between 3-Gly33 and 8-Gly33 (**D**_**HC**_). B. Distance from Phe19 to Val39 (**D**_**VC**_). C. Distance between 3-Gln15 and 8-Gln15 (**D**_**HE**_). D. Distance from Glu11 to Val39 (**D**_**VE**_). E. Distance between His14 of each core strand (**D**_**IS**_).

To further analyze the changes induced by mutations in the tertiary structure of the fibers, and as done in our previous study,^30^ the same five-distances analysis was applied to measure the compactness of the fiber (see Figure 6):

1 Horizontal measure in the core region **(D**_**HC**_**)**: the distance between 3-Gly33 and 8-Gly33 (Figure 6A) shows the fiber opening of the core region in the horizontal X-axis and indicates if the β-sheet interaction is weakened -longer distance- or strengthened -shorter distance-.
2 Vertical measure in the core region **(D**_**VC**_**)**: the average distance from Phe19 to Val39 (Figure 6B) of each core strand shows the opening along the vertical Y-axis, measuring the compactness of the S-shaped supramolecular structure.
3 Horizontal measure in the external region **(D**_**HE**_**)**: the distance between 3-Gln15 to 8-Gln15 (Figure 6C) indicates how the fiber opens horizontally with respect to the N_Ter_ region, a representation of the β-sheet interaction at this region.
4 Vertical measure in the external region **(D**_**VE**_**)**: the average distance from Glu11 to Val39 (Figure 6D) of each core strand describes how the N_Ter_ tail separates from the core region of the fiber.
5 Interstrand measure **(D**_**IS**_**)**: the average distance between His14 of each core strand (Figure 6E) differentiates between collective or individual fiber movement.

**Figure 7:**
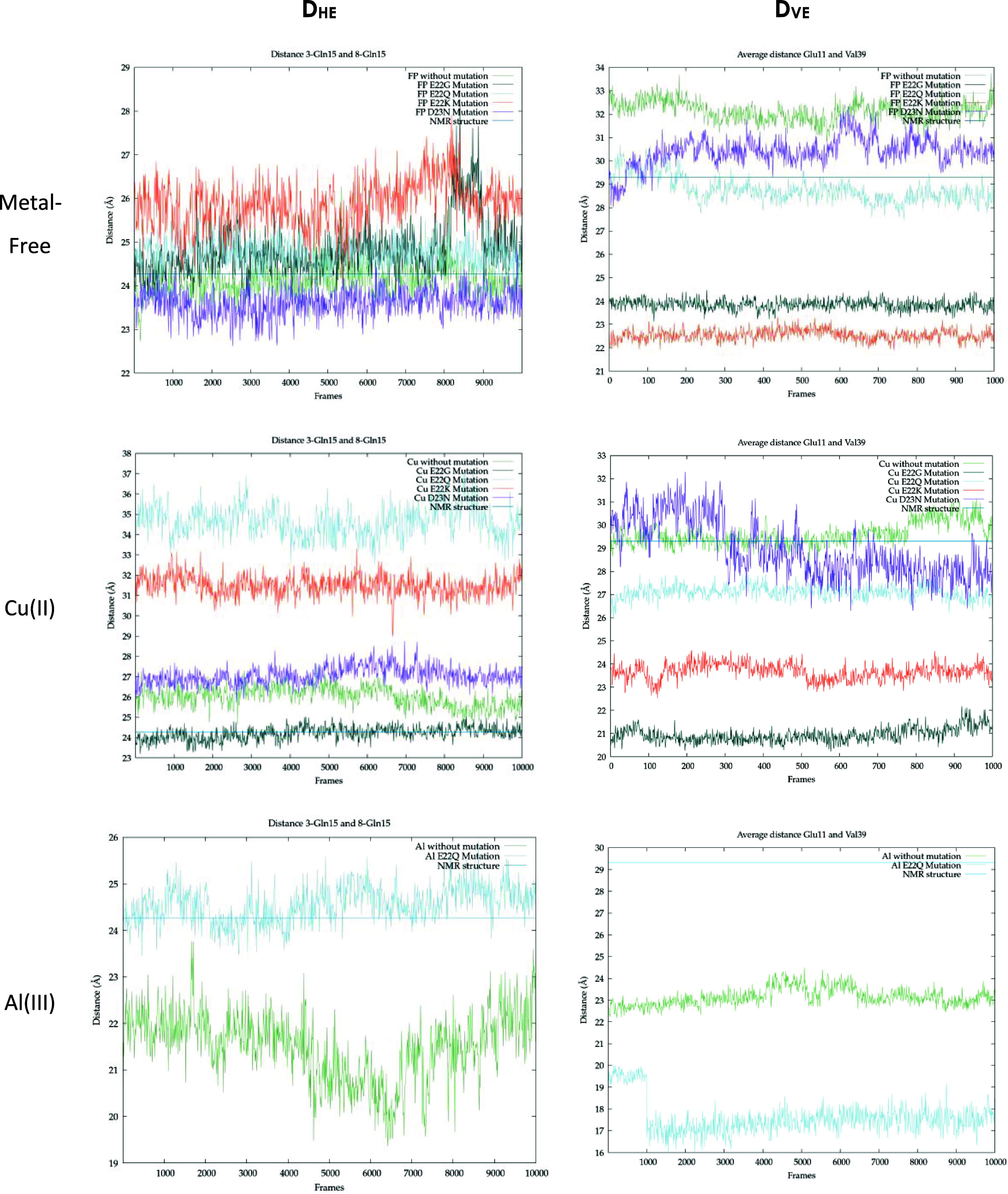
Metal-free, Cu(II), and Al(III) bound distances of the 3-GLN15 8-GLN15 measure (**D**_**HE**_) and GLU11-VAL39 measure (**D**_**VE**_) for WT (light green), E22Q (light blue), E22G (dark green), E22K (orange), and D23N (purple) variants.

The analysis of all distances indicates that those corresponding to the core of the fiber (**D**_**HC**_ and **D**_**VC**_) do not change significantly upon mutation. However, those involving residues at the most external part of the fiber suffer more significant changes (**D**_**HE**_ and **D**_**VE**_), mainly when bound to metal ions, and thus, only values for these descriptors are reported in Figure 7. The remaining ones are given in the SI (Figures S20, S25, and S27).

**Figure 8:**
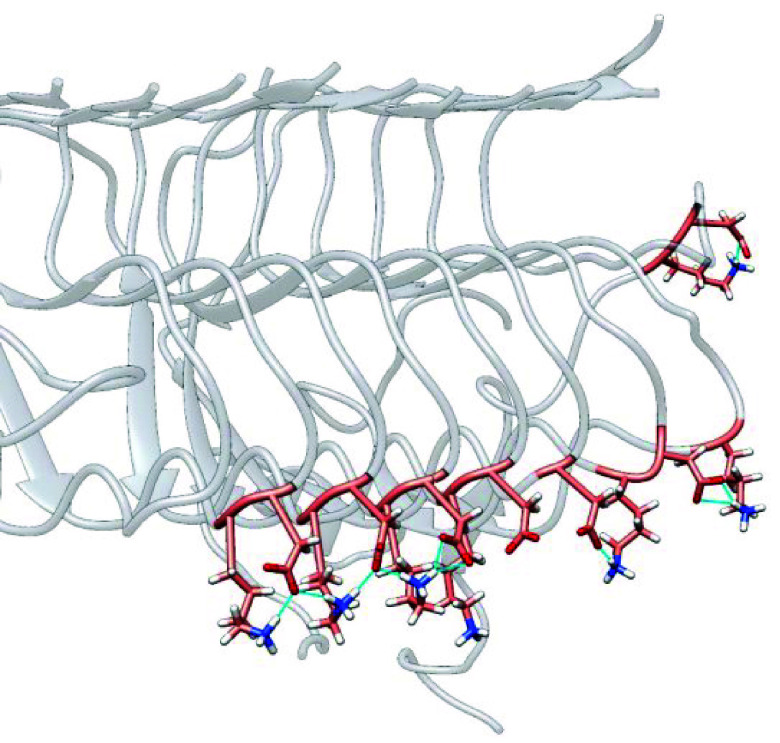
Hydrogen bonds (blue line) formed between Lys22 and Asp23 in the initial structure of the E22K MD simulation.

For metal-free fibers, the most remarkable result is observed for the most representative distance in the fan-like movement, **D**_**VE**_ (Glu11-Val39), which shows a significant decrease in E22K (22.6 Å), E22G (23.8 Å), E22Q (28.7 Å), and D23N (30.4 Å) compared to the WT (32.1 Å) (Figure 7). Thus, the four mutations tend to reduce the system’s flexibility and stabilize the S-shape of each monomer by reducing the fan-like movement. This reduction of the breathing movement could be attributed to the fact that the mutations substitute a negatively charged residue (E) with a neutral residue (Q and G) or a positively charged (K) one, which reduces the repulsion with D23. Furthermore, E22K allows the formation of strong hydrogen bonds between Lys22 and Asp23 (Figure 8) (mainly of the same strand), and E22Q allows the formation of mild hydrogen bonds between Gln22 and Asp23, which hinder such movement.

**Figure 9:**
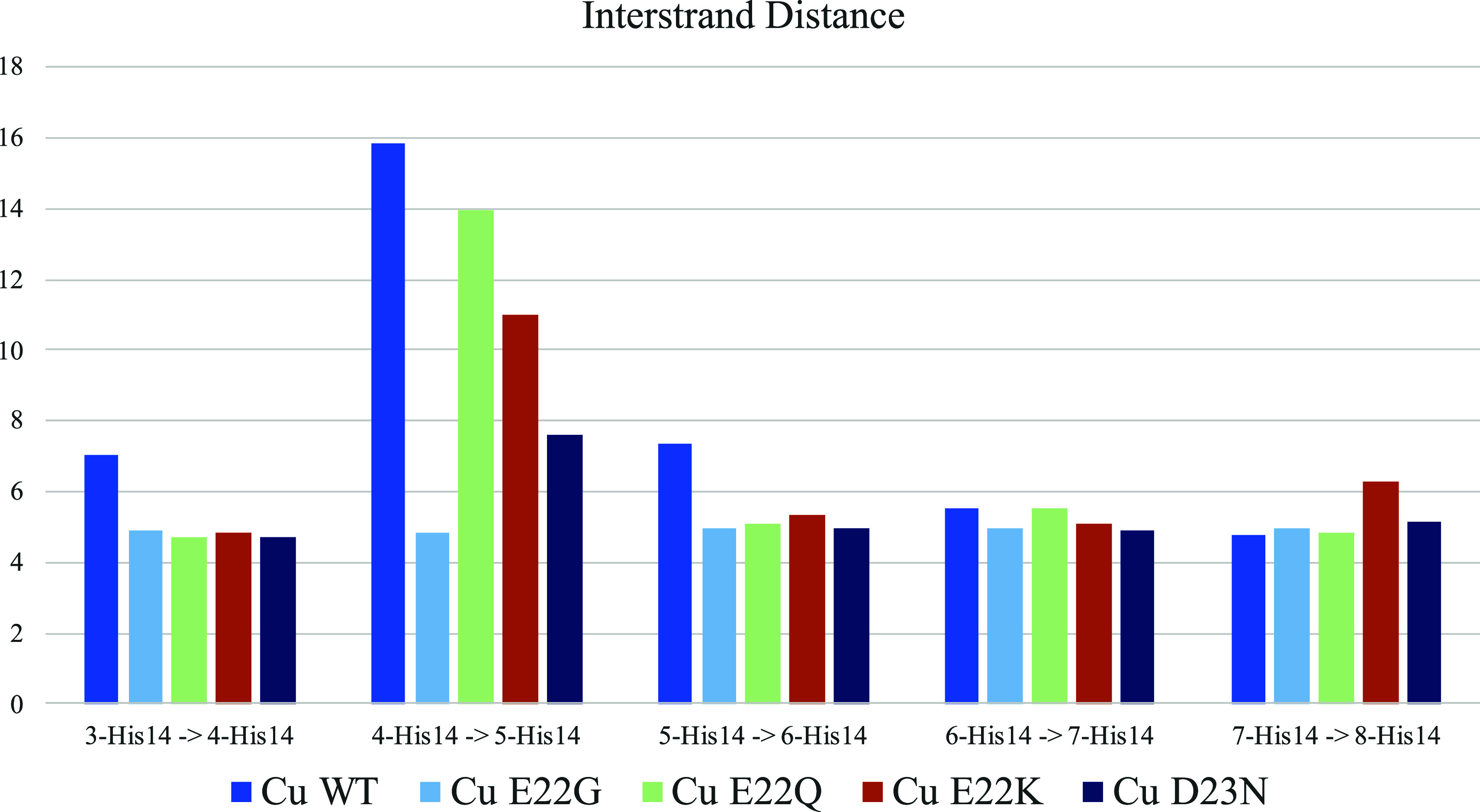
Interstrand distance (**M**_**IS**_) in Cu(II) bound systems.

With respect to the Cu(II)-bound aggregate, both the fan-like movement and the intrinsically disordered behavior of the N_Ter_ region (residues 1 to 11) are observed among the variants (Figure 5). However, the fan-like movement is reduced for all mutants compared to the WT (see Figure 7) since the **D**_**VE**_ values (21–28 Å) for Cu(II) mutants are smaller than that of the Cu(II)-WT (29–30 Å). Indeed, the formation of hydrogen bonds for Cu-E22K and Cu-E22Q systems is also observed, as in the metal-free systems. On the other hand, for Cu(II)-E22Q and Cu-E22K, the horizontal breathing motion increases the **D**_**HE**_ distance up to 35 and 31 Å, respectively (Figure 7), which is much larger than that observed for Cu(II) bound WT, E22G, and D23N variants (24–27 Å). Interestingly, in our previous study, we observed that under Cu(II) binding to the WT some strands start to dissociate in the course of the simulation, thereby suggesting a possible disruption of the aggregate, as the **D**_**IS**_ distance between 4-His14 and 5-His14 increases significantly (Figure 9). This effect is absent in the Cu(II)-E22G systems and is slightly reduced for Cu(II)-E22Q, Cu(II)-E22K, and Cu(II)-D23N systems, suggesting that the mutations’ effect compensates for the destabilizing effect of the metal binding. Remarkably, Cu(II)-E22Q and Cu(II)-E22K variants show large distances for both **D**_**HE**_ and **D**_**IS**_, while Cu(II)-WT demonstrates an increased **D**_**IS**_ magnitude. This is related to the fact that the disruption observed between strands 4 and 5 is not considered by the **D**_**HE**_ measure, which measures the global distance between strands 3 and 8.

Altogether, the analysis of the four familial variants shows the mutations’ impact at different levels on the aggregate structure. Associated with the binding of Cu(II), mutations tend to improve the stability of the S-shape form of the fiber, although destabilizing the interstrand interactions, except for Cu(II)-E22G, which retrieves a more compact system. The higher interstrand stability caused by Cu(II)-E22G may be related to the fact that changing Glu to Gly reduces the size of the lateral chain of the residues and, hence, the hydrophobic repulsion.

Regarding the Al(III) bound systems, only one mutation could be studied, corresponding to the Al(III)-E22Q variant since metal coordination occurs with residues 22 and 23, and in all other variants, the introduced residues are not capable of coordinating Al(III). For Al(III)-E22Q, molecular dynamics simulations show a slight disruption of the lateral strands related to the fan-like movement, also visible in the Al(III)-WT form (Figure 5). Concerning the measures, the effect of the E22Q mutation on the Al(III) bound systems is consistent with that already observed for Cu(II), increasing **D**_**HE**_ up to 25 Å but reducing **D**_**VE**_ up to 17 Å (Figure 7). Thus, such a variant consistently increases the breathing movement in the horizontal axis, while the S-shape is further stabilized. Besides, the structural organization of the N_Ter_ region in the Al(III)-E22Q complex is maintained similarly to the WT system.

## Conclusions

This study analyzes the dynamic behavior of four of the most relevant familial mutations of AD patients in both monomeric and aggregate forms of Aβ_42,_ in the absence and presence of Cu(II) and Al(III) metal cations.

Regarding the metal-free monomers, all of the mutations have a similar effect, leading to a decrease of the α-helix content. Besides, the β-sheet content substantially increases for E22K and E22G (39% and 7%, respectively). Since the β-strand structure is a significant marker of the fibril, these results suggest that these mutations could favor the formation of well-organized aggregates. When considering familial mutations and metal binding, the metal effect seems prevalent in mutations, inducing the formation of U-shaped structures stabilized by interhelical contact. The only exception is Cu(II)-E22G, for which the U-shaped structure does not appear due to the formation of β-sheets maintained throughout the 1 μs GaMD simulation. The presence of β-sheet content is also observed for Al(III)-E22G, though with less prevalence. This agrees with glycine being a known helix breaker, which leads to lower percentages of the U-shape among metal-bound mutated structures. Differences between the Cu(II) and Al(III) systems can be related to the differences in metal coordination, which affects the distribution of α-helices in the monomer.

Regarding the impact of the familial mutations on the fibrillar structures, the effect observed of metal binding is consistent with that reported in our previous study of the WT-Aβ_42_ fibrillar form,^30^ since Cu(II) interactions at the N_Ter_ region of the fiber disrupt the characteristic tertiary structure of the fiber. In contrast, Al(III) binding further stabilizes the S-shape. However, the effect of the amino acid substitutions seems to prevail over those related to metal-binding as consistent trends are observed along systems regardless of the metal-binding state. E22Q and E22K mutations decrease the opening of the fiber along the vertical axis. Such reduction of the fan-like breathing movement could be attributed to the formation of strong and mild hydrogen bonds between Lys22 and Gln22 with Asp23, respectively, together with the reduction of the overall charge of the system. The E22G mutation seems to further stabilize the fibrillar structure, primarily by reducing its disorganization and leading to more compact fibers, in both the vertical and horizontal axes, which is attributed to the reduction of the negative charge and the absence of a lateral chain at residue 22. These changes could be responsible for the differential aggregation rates observed for E22 mutations.^2,4^ Lastly, the D23N mutation reduces the system’s stability, likely due to the abolishment of the D23-K28 salt bridge, leading to the most flexible complexes of the entire set and involving motions both in the horizontal and vertical axes of the aggregates. Such results correlate well with experimental observations in which the D23N fiber is less thermodynamic and kinetic stable.^33^

## Methods

### Initial Models

Several models are available for the monomeric structure of the Aβ peptide (PDB codes 1IYT, 1Z0Q, 2LFM). However, the 1IYT structure is obtained in an apolar environment, which does not represent the biological environment, while 2LFM is obtained in an aqueous solution in the presence of a salt. For 1Z0Q, a very low punctuation in clashscore, Ramachandran outliers, and side chain outliers is obtained. Hence, the original structure of the monomeric peptide used in this study corresponds to the one available in the Protein Data Bank with the accession code 2LFM structure, with better overall punctuation in the Protein Data Bank.^37^ As this structure is 40 amino acids long, the full 42 residues long species and all the mutations (E22G, E22Q, E22K, and D23N) were generated using UCSF Chimera Software.^38^

**Scheme 1:**
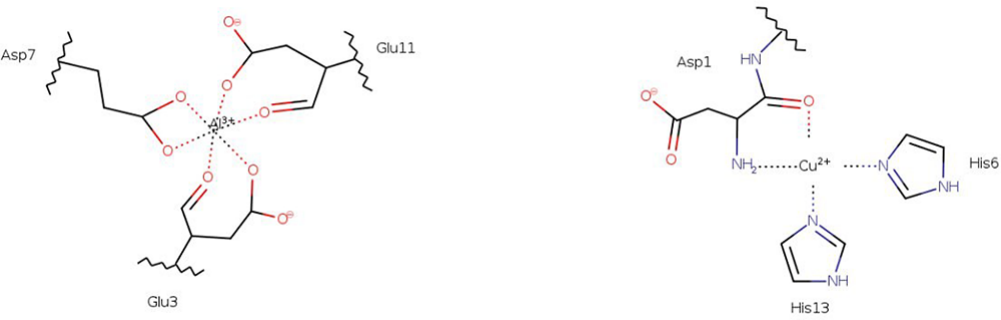
Representative Coordination Mode of Al(III) and Cu(II) with Monomeric Aβ

The metal binding spheres for the monomeric species were chosen according to the previous works of Alí-Torres and Mujika.^22,23,39^ For Al(III), we selected the Al_I_1_ coordination, which involves up to three carboxylate groups from Glu3, Asp7, and Glu11 (Al-complexes hereafter). At the same time, for Cu(II), we considered Cu_I_2_, involving His6, His13, and both N_Ter_ and CO from Asp1 in the coordination sphere,^12^ as representative of the most stable structures (see Scheme 1) (Cu-complexes hereafter).

Several crystallographic structures are available for the fibrillar form of Aβ (PDB codes 2NAO, 5OQV, and 2MXU). Though the 2NAO structure corresponds to the whole 1–42 sequence, it retrieves poor punctuation in all the PDB parameters (clashscore 22, Ramachandran 12.4%, and Sidechain outliers 26.7%), while the 2MXU PDB structure retrieves far better punctuation (clashscore 0, Ramachandran 3.8%, and side chain outliers 6.22%). With respect to 5OQV, such a structure corresponds to two intertwined protofilaments, representing a higher aggregation state in which the quaternary structure is observed, with residues 1–11 being in an organized conformation due to the formation of hydrogen bonds between Asp1 and Lys28 from opposite fibers. Hence, the most adequate study for the initial fibrillar forms is the Protein Data Bank 2MXU structure,^40^ which was used for building the fibrillar models, comprising residues 11 to 42. Accordingly, the missing region was modeled using Homology Modeling.^41^ For Cu(II) bound systems, the coordination sphere was already established in our first paper on metal-fiber coordination,^30^ applying BioMetAll software^42^ and Gold software,^43^ whose parameter file was modified to include atom types for metal ions and their possible coordinating amino acids.^44^ The binding sites found were parametrized through the MCPB.py protocol,^45^ from quantum mechanical calculations with DFT(B3LYP) and adding Grimme’s correction for dispersion.^46^ The 6–31+G(d,p) basis set^47^ was used for all atoms, while Seminario’s method^48^ and RESP calculations^49^ were applied to obtain force constants and point charges, respectively.

### Classical Molecular Dynamics Simulations

Molecular Dynamics simulations were performed with the AMBER ff14SB force field.^50^ The parameters for the metallic environment were obtained using the Amber tool MCPB.py.^45^ The complex obtained was embedded into a cubic box of TIP3P water molecules, neutralizing the charge with Cl^–^ and Na^+^ (Table 4).

**Table 4:** Number and Type of Ions Added to Neutralize the Systems

	WT	E22G	E22Q	E22K	D23N	Cu(II)-WT	Cu(II)-E22G	Cu(II)-E22Q	Cu(II)-E22K	Cu(II)-D23N	Al(III)-WT	Al(III)-E22G	Al(III)-E22Q	Al(III-E22K	Al(III)-D23N
Monomer	3 Na^+^	2 Na^+^	2 Na^+^	1 Na^+^	2 Na^+^	2 Na^+^	1 Na^+^	1 Na^+^	0	1 Na^+^	0	1 Cl^–^	1 Cl^–^	2 Cl^–^	1 Cl^–^
Fiber	30 Na^+^	20 Na^+^	20 Na^+^	10 Na^+^	20 Na^+^	20 Na^+^	10 Na^+^	10 Na^+^	0	10 Na^+^	15 Na^+^	X	5 Na^+^	X	X

First, a 10 ns MD simulation in the NPT ensemble with a one fs integration time step was performed. Constant temperature and pressure were set with a Langevin thermostat at 300 K and a barostat at 1.01325 bar, respectively. The hydrogen atom bonds were constrained with the SHAKE algorithm. This first simulation was intended to relax the system before the GaMD simulation.

### Gaussian accelerated Molecular Dynamics Simulations

Gaussian accelerated Molecular Dynamics (GaMD)^51^ allows an enhanced conformational exploration, as the harmonic boost potential applied to the system prevents it from getting stuck in local minima. For monomeric systems, 1 μs GaMD simulations were performed, starting from the last point of the previous MD simulation. Three GaMD of 1 μs each was performed for fibrillar ones to generate replicas and ensure an optimal exploration. The force field was maintained for the GaMDs in an NVT ensemble, again constraining the hydrogen atom bonds with SHAKE.^52^ The integration time step used in this second simulation is 2 fs. The boost is applied to dihedral and total potential energy (igamd = 3).

### Exploration Analyses

The free energy profile was computed using the Generalized Born Implicit Model,^53^ for 800 frames -monomeric- and 1000 frames -fibrils- extracted along the trajectories, stripping water molecules and applying a short minimization prior to the energy calculation. The energy profile is represented together with the Radius of Gyration along the trajectory, computed with CPPTRAJ,^54^ which informs about the systems’ compactness. For all systems, another MD of 100 ns was performed on the lowest energy well extracted from the implicit solvent calculation (see below) of the GaMD. All of the following analyses are performed over such trajectories. The proper exploration of the systems was assisted through several criteria, including the PCA analysis,^55^ and the RMSD all-to-all of the trajectory and countering cluster.

### Monomers

Contact Maps were obtained for the monomeric structures with the Contact Map Explorer package for MDtraj. To analyze the secondary structure of the system, several parameters were evaluated. First, the α-helix content was computed together with the locations of the turn residues. The percentage of the U-shaped pattern was computed by clustering the whole MD trajectory and considering the number of frames composing each cluster. Then, the U-shaped percentage was computed by dividing the number of structures demonstrating the U-shaped pattern with respect to the whole number of frames in the trajectory. Hydrogen Bond (HB) contacts in the 16–42 region were also considered with regard to the most important contacts for structuring the peptide. Finally, the mean radius of the gyration was also computed.

### Fibers

First of all, the PCA movements of the fibers along the trajectories were extracted with the VMD tool NMWizard counterchecked by Normal Mode Analysis performed with WEBnma software,^56−58^ to study the global low energy internal motions of the systems.

## Supporting Information Available

Energy profiles, timeline, and stability analysis are provided for the monomeric species. Energy profiles, stability analysis, and distance measurements are available for the fibrillar species
  (PDF)
