# Supplementary material for: Computational Study of Amyloidβ42 Familial Mutations and Metal Interaction: Impact on Monomers and Aggregates Dynamical Behaviors
Source: Inorg Chem. 2024 Feb 26;63(10):4725–37. doi: 10.1021/acs.inorgchem.3c04555 (PMC10934806; doi:10.1021/acs.inorgchem.3c04555)
Supplement: Supplementary file 1 — ic3c04555_si_001.pdf [file ic3c04555_si_001.pdf]

Supporting Information: Computational study of Amyloidb$_{42}$ familial mutations and metal-interaction: Impact on monomers and aggregates dynamical behaviors.

Lorena Roldán-Martín,[a] Mariona Sodupe,*,[a] Jean-Didier Maréchal *,[a]

[a]Departament de Química, Universitat Autònoma de Barcelona, 08193, Cerdanyola del Vallès, Spain

*mariona.sodupe@uab.cat, jeandidier.marechal@uab.cat

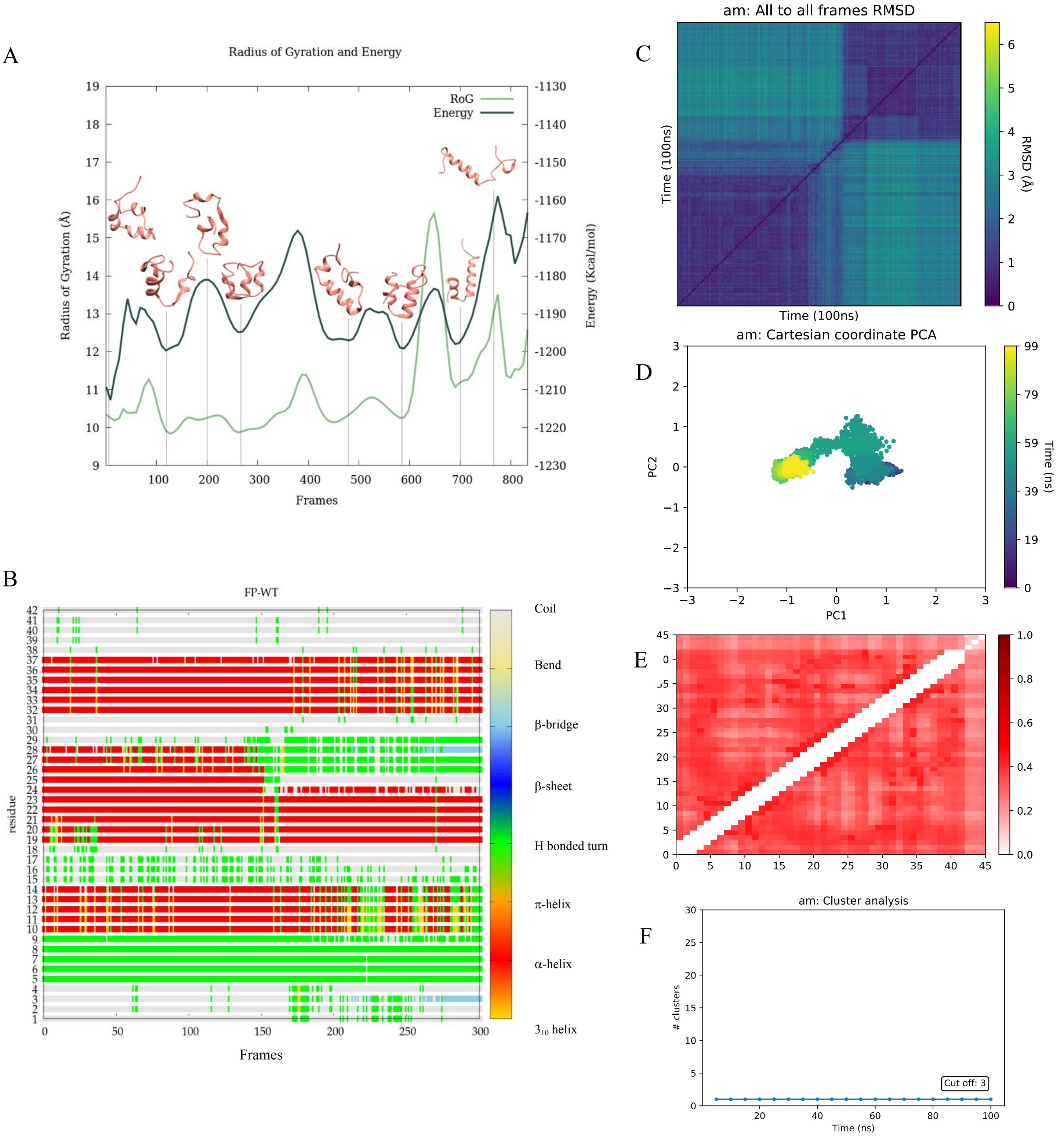

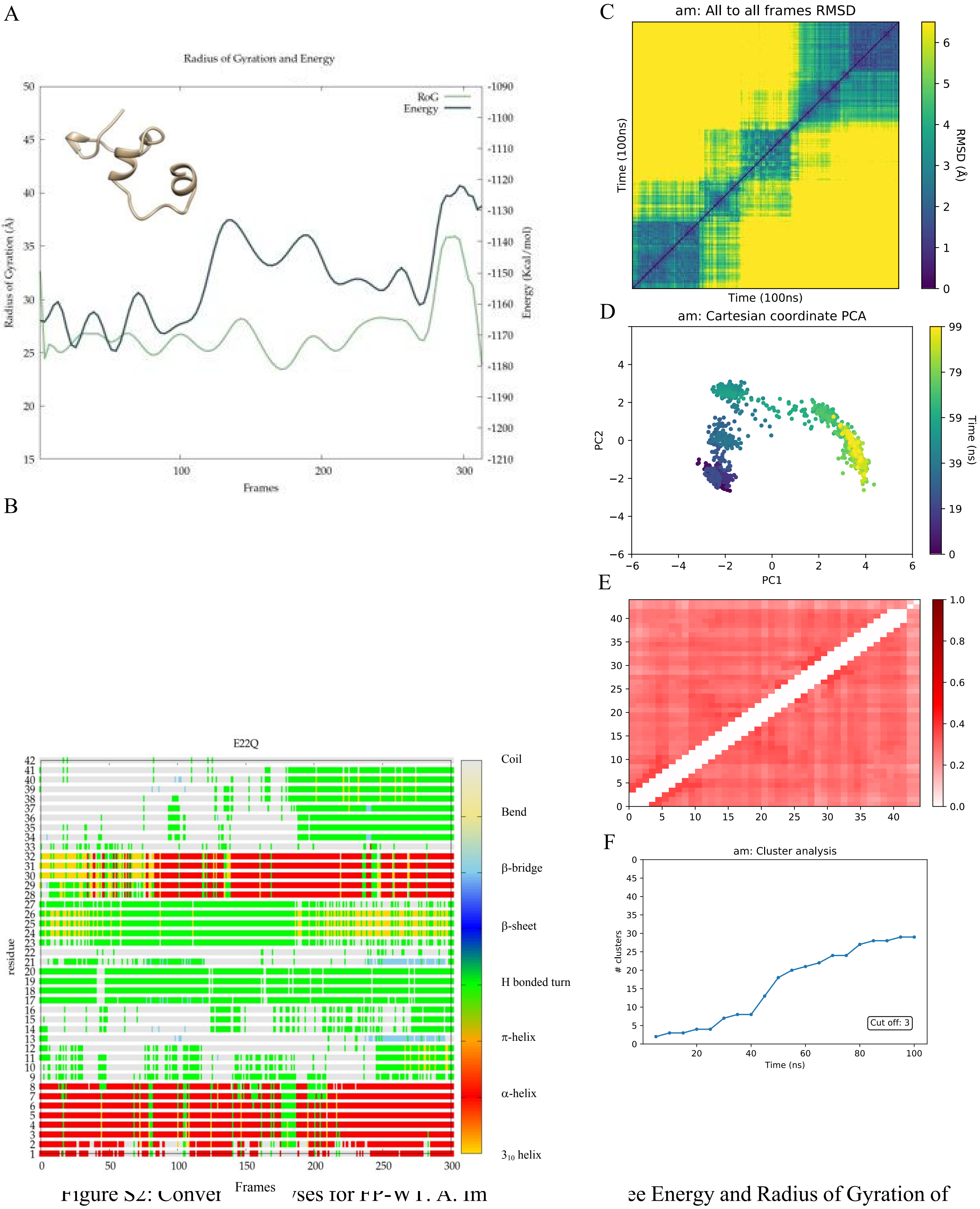

Figure S2: Conver[illegible]yses for FP-WT. A: Tim[illegible]ee Energy and Radius of Gyration of the GaMD simulation, with the lowest energy structure. Timeline Analysis (B), RMSD all to all (C), PCA exploration (D), Contact Map (E) and Cluster Countering (F) of the MD simulation.

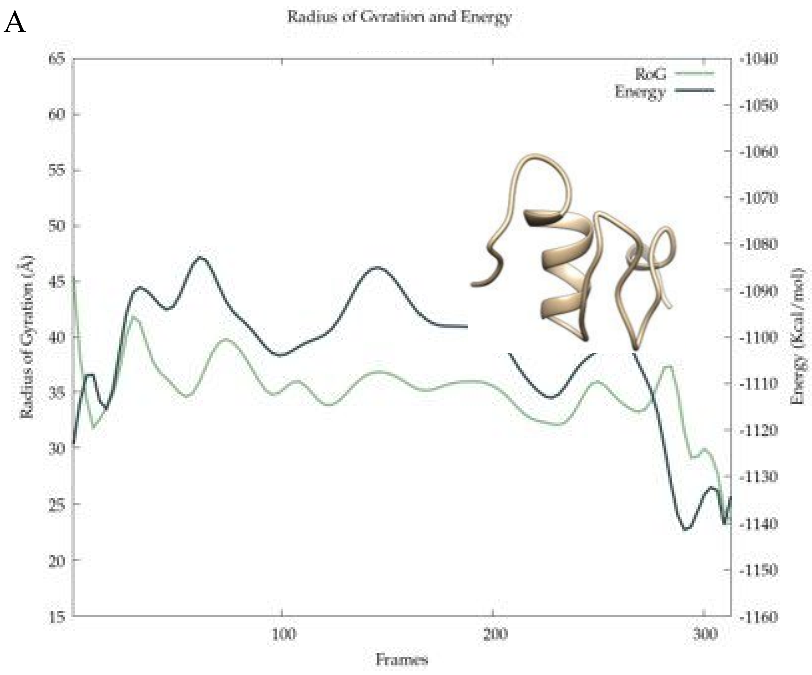

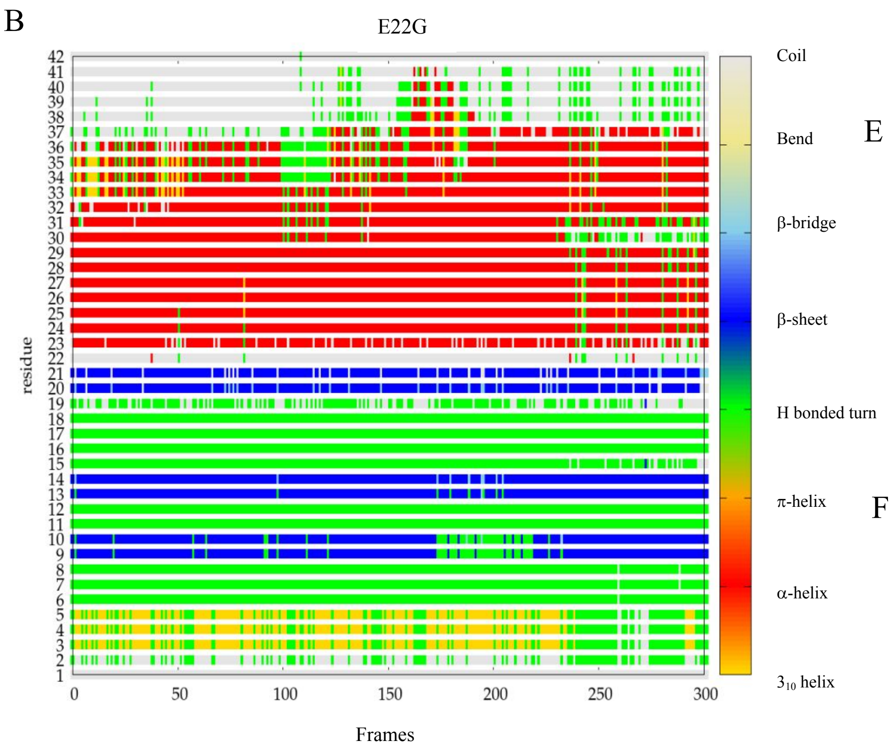

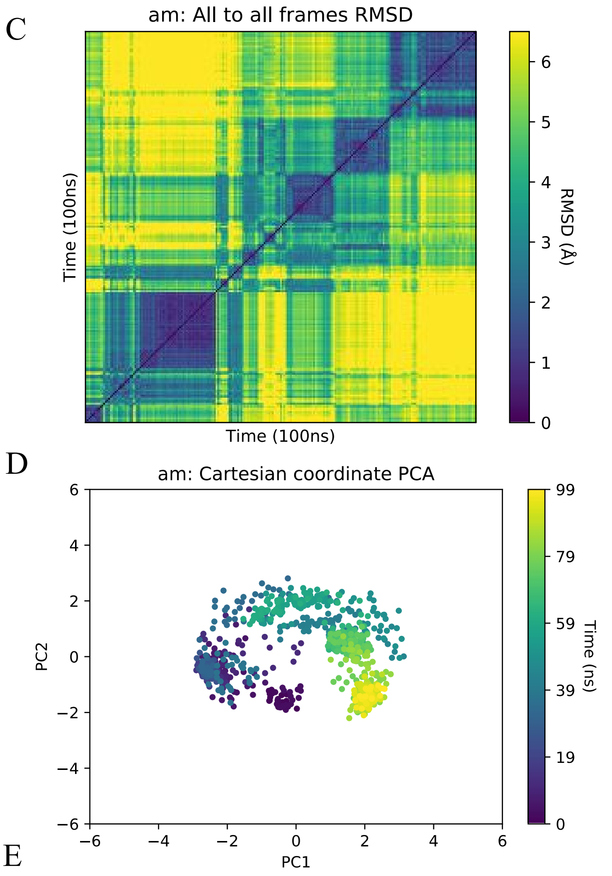

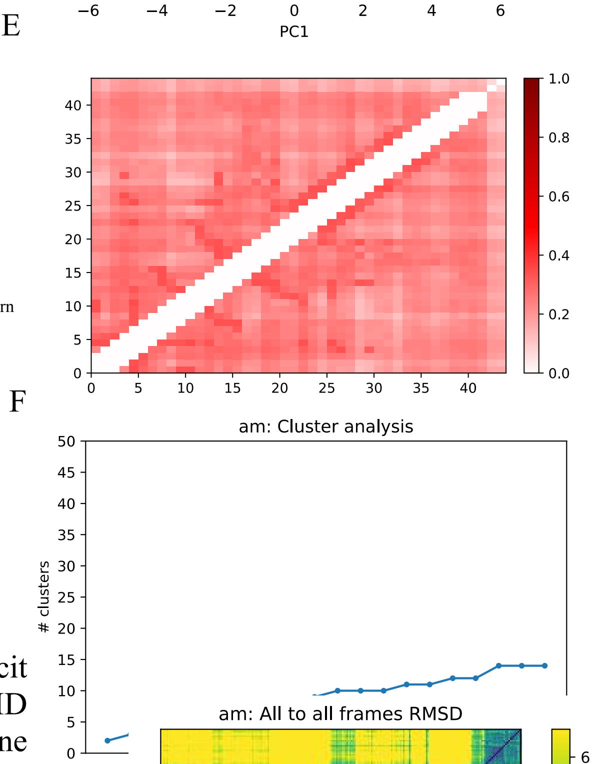

Figure S3: Converge analyses for FP-E22G. A. Implicit solvent Free Energy and Radius of Gyration of the GaMD simulation, with the lowest energy structure. Timeline Analysis (B), RMSD all to all (C), PCA exploration (D), Contact Map (E) and Cluster Countering (F) of the MD simulation.

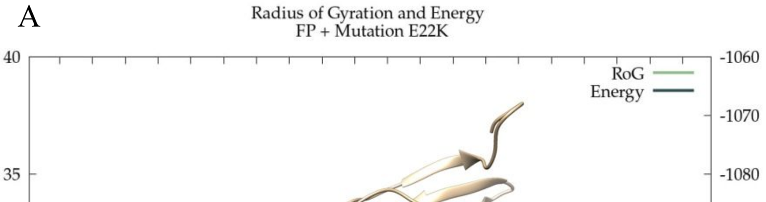

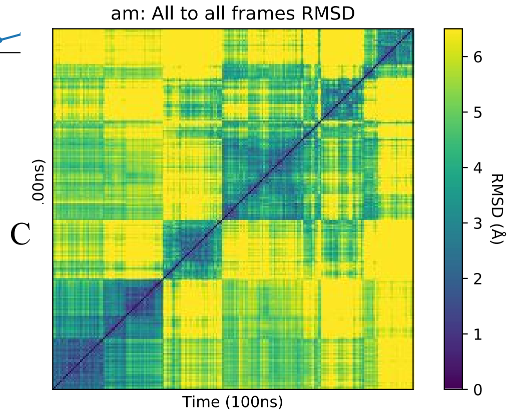

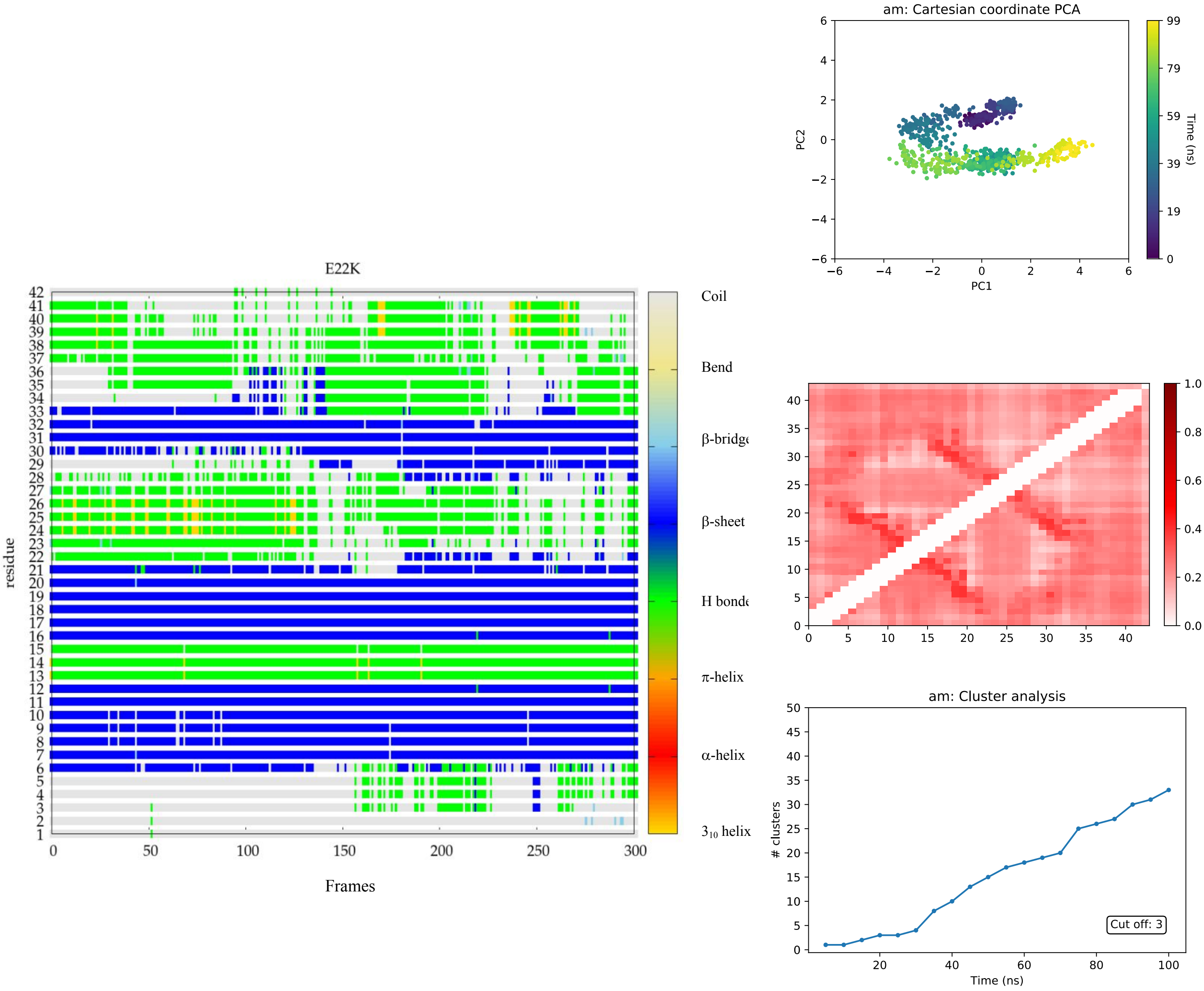

Figure S4: Converge analyses for FP-E22K. A. Implicit solvent Free Energy and Radius of Gyration of the GaMD simulation, with the lowest energy structure. Timeline Analysis (B), RMSD all to all (C), PCA exploration (D), Contact Map (E) and Cluster Countering (F) of the MD simulation.

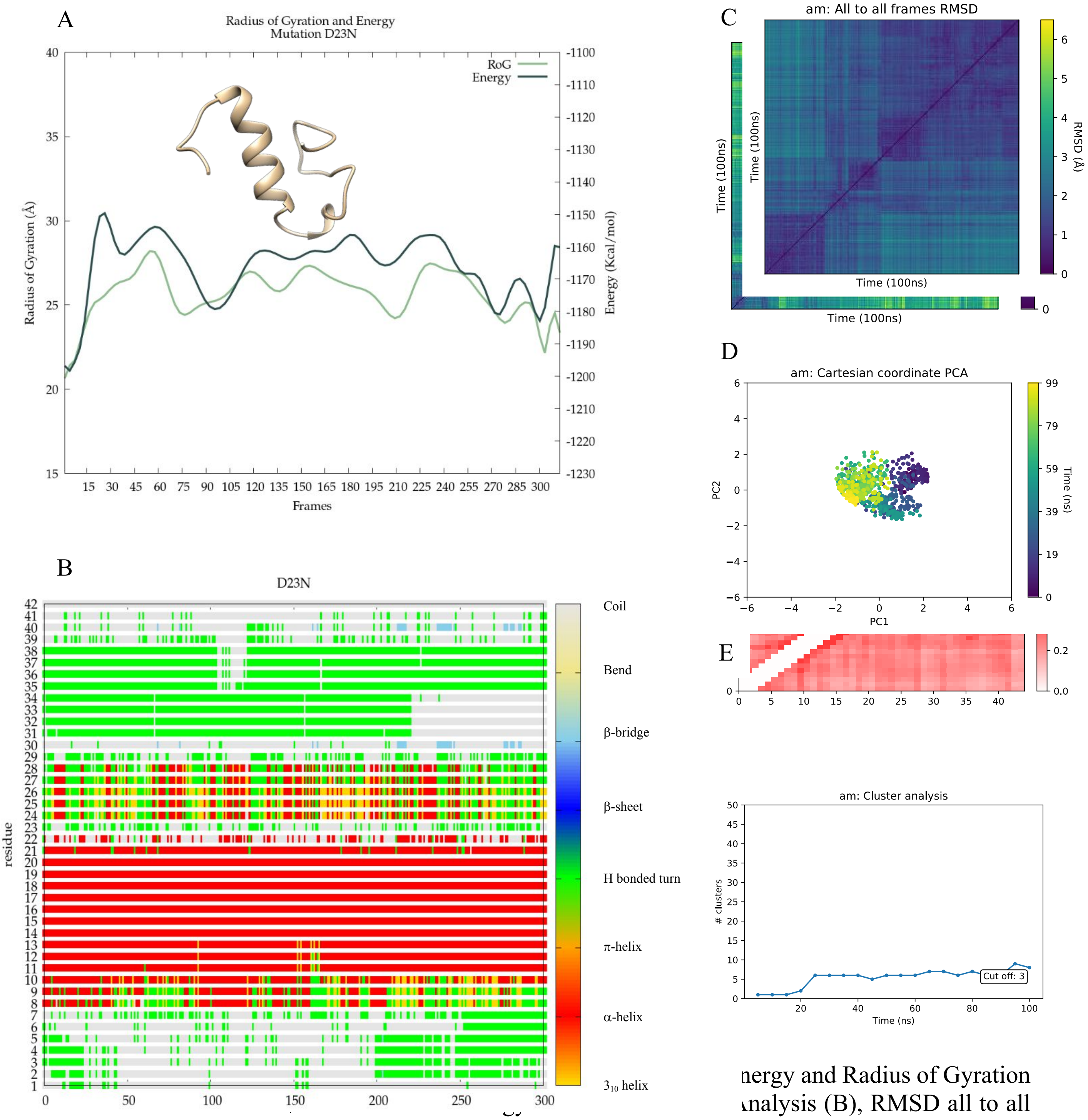

nergy and Radius of Gyration
nalysis (B), RMSD all to all
(C), PCA exploratio ontact Map (E) and Cluster Countering (F) of the MD simulation.

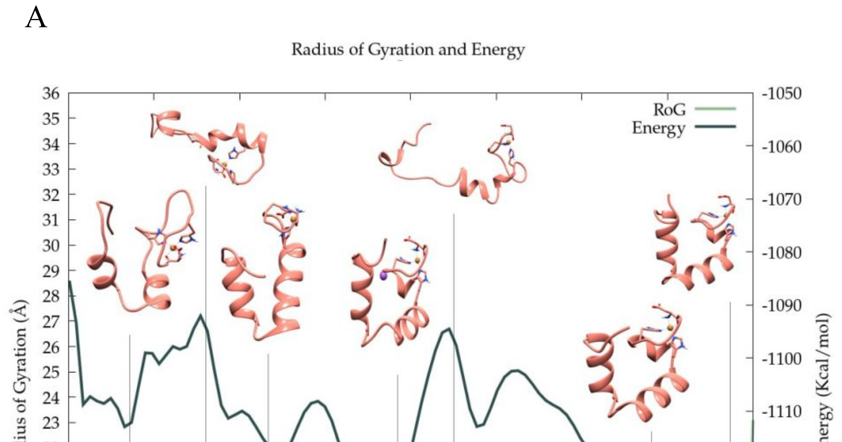

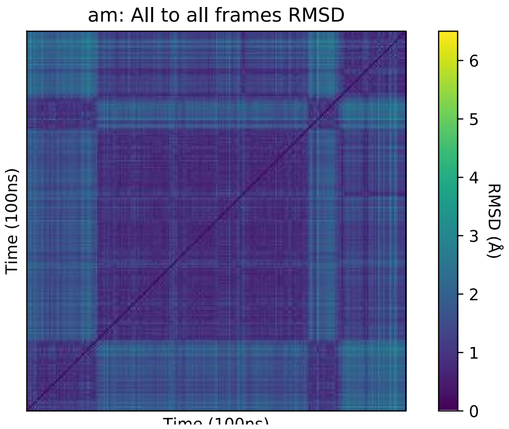

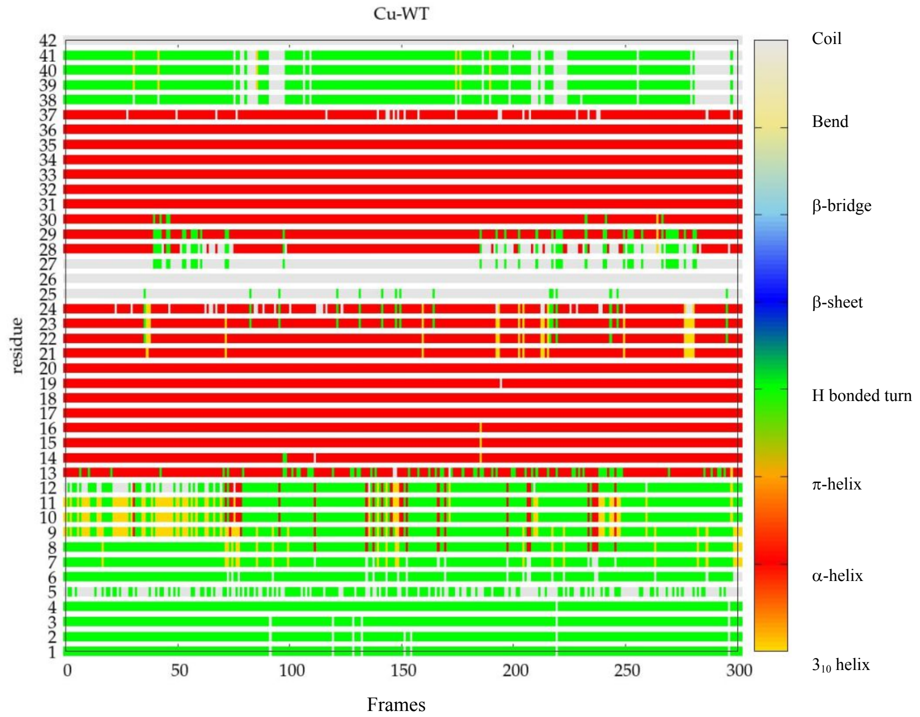

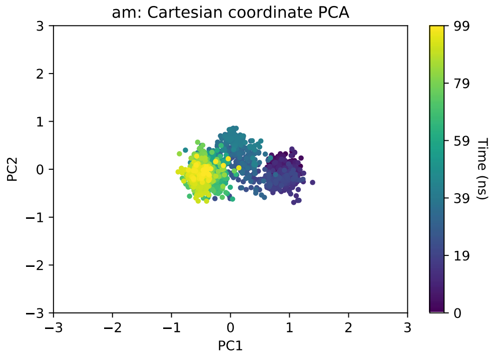

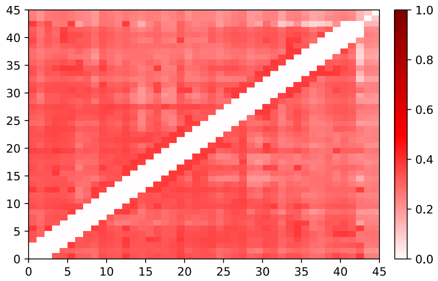

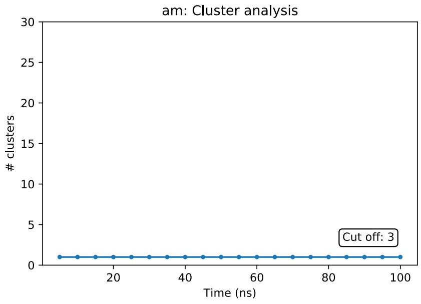

Figure S6: Converge analyses for Cu-WT. A. Implicit solvent Free Energy and Radius of Gyration of the GaMD simulation, with the lowest energy structure. Timeline Analysis (B), RMSD all to all (C), PCA exploration (D), Contact Map (E) and Cluster Countering (F) of the MD simulation.

A

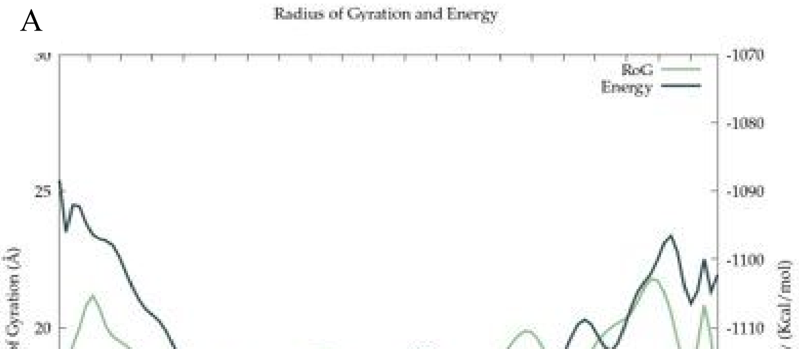

C

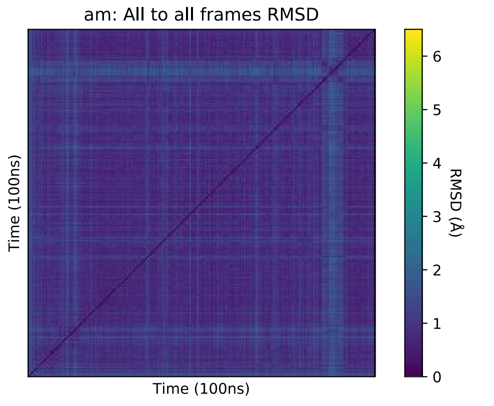

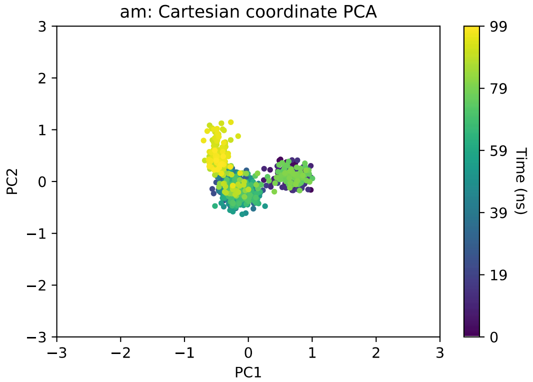

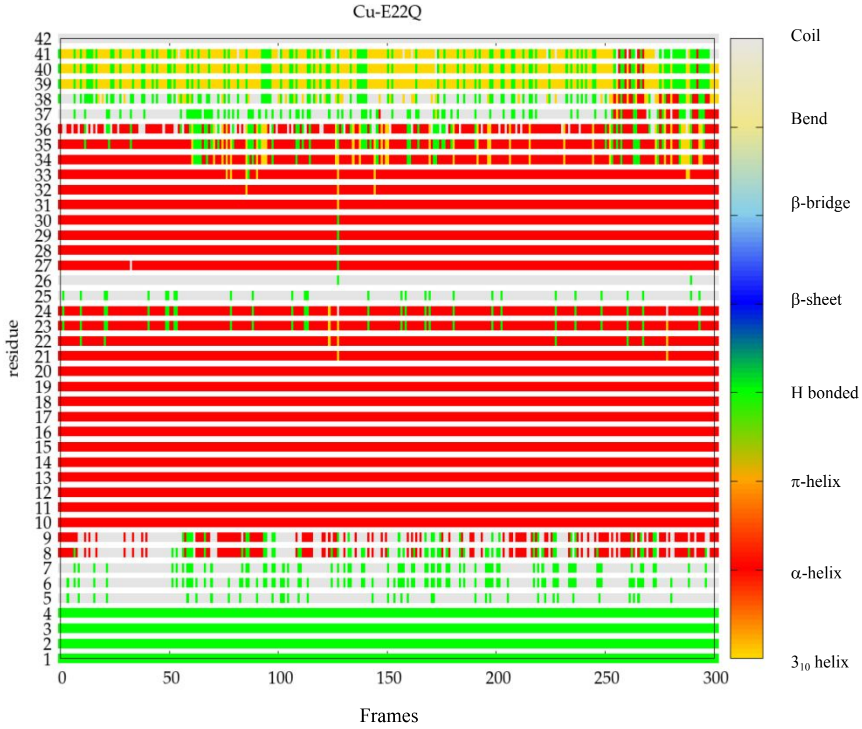

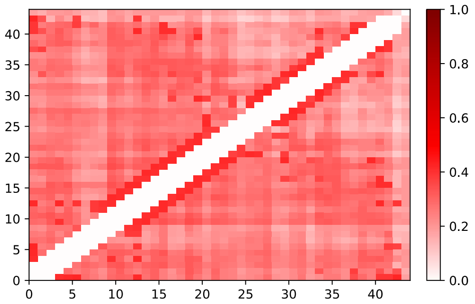

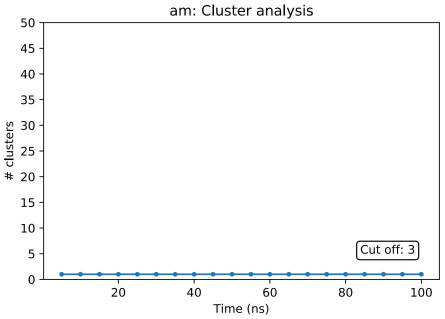

Figure S7: Converge analyses for Cu-E22Q. A. Implicit solvent Free Energy and Radius of Gyration of the GaMD simulation, with the lowest energy structure. Timeline Analysis (B), RMSD all to all (C), PCA exploration (D), Contact Map (E) and Cluster Countering (F) of the MD simulation.

A

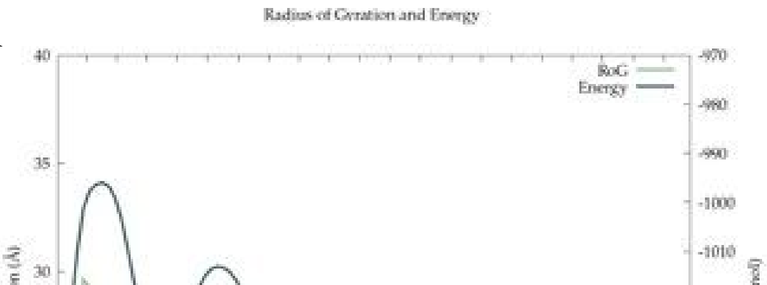

C

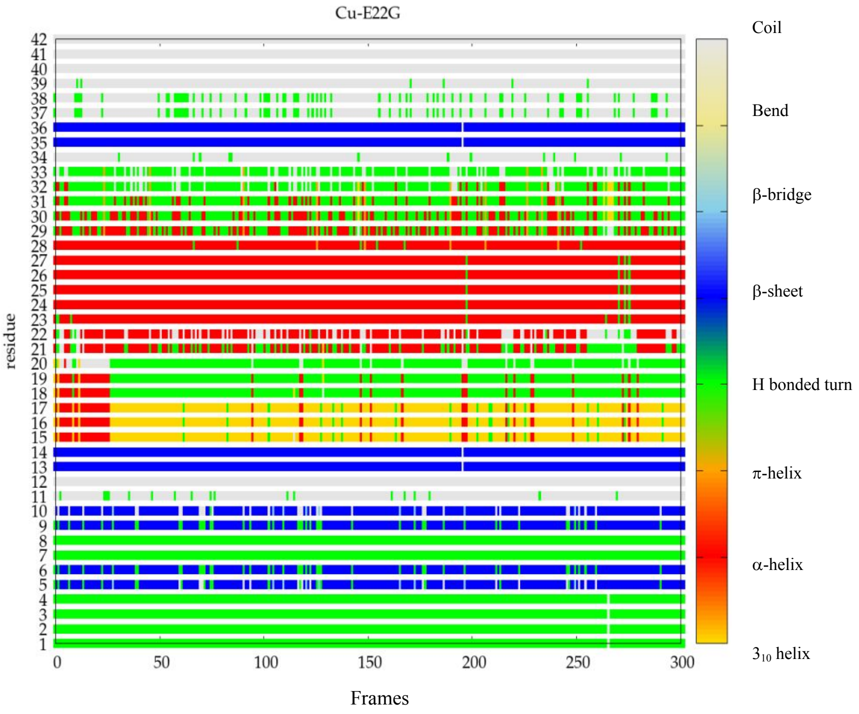

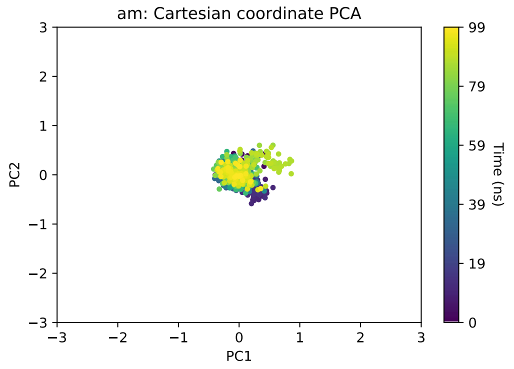

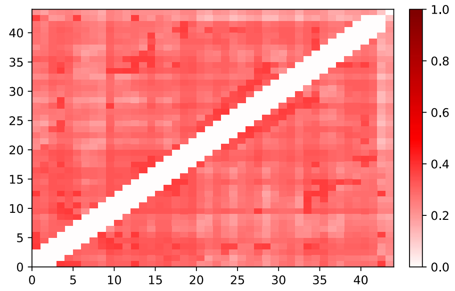

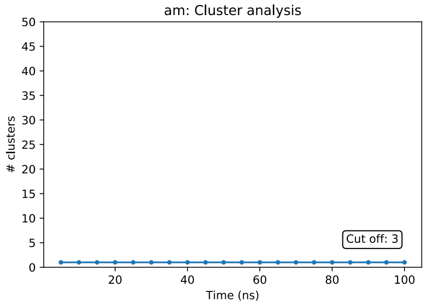

Figure S8: Converge analyses for Cu-E22G. A. Implicit solvent Free Energy and Radius of Gyration of the GaMD simulation, with the lowest energy structure. Timeline Analysis (B), RMSD all to all (C), PCA exploration (D), Contact Map (E) and Cluster Countering (F) of the MD simulation.

A

C

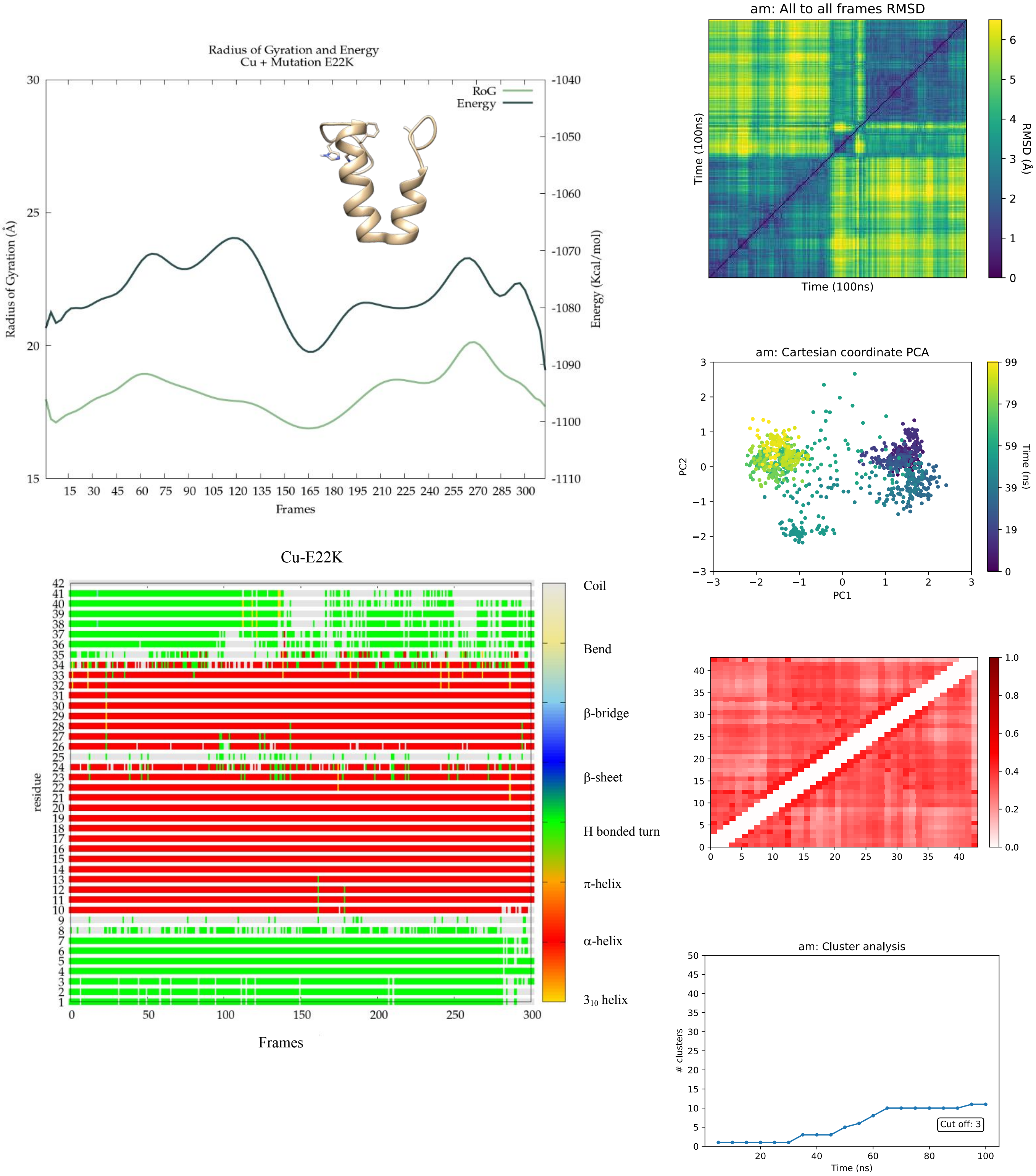

Figure S9: Converge analyses for Cu-E22K. A. Implicit solvent Free Energy and Radius of Gyration of the GaMD simulation, with the lowest energy structure. Timeline Analysis (B), RMSD all to all (C), PCA exploration (D), Contact Map (E) and Cluster Countering (F) of the MD simulation.

A

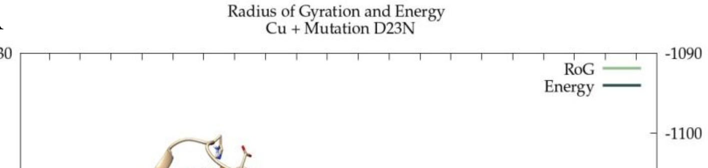

C

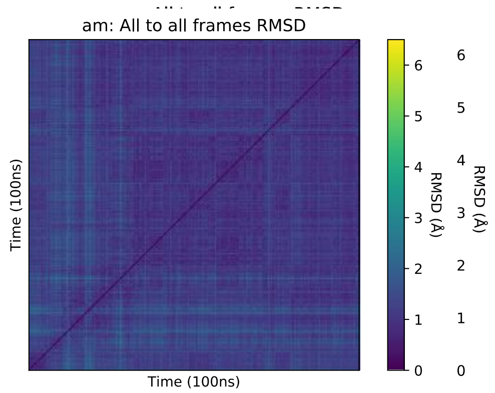

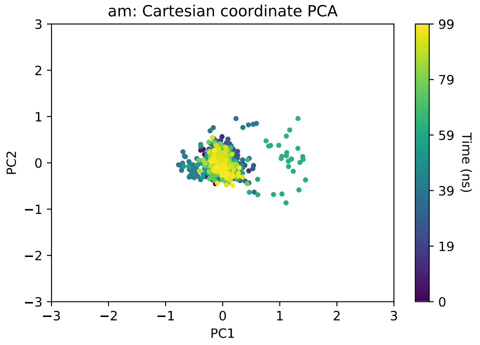

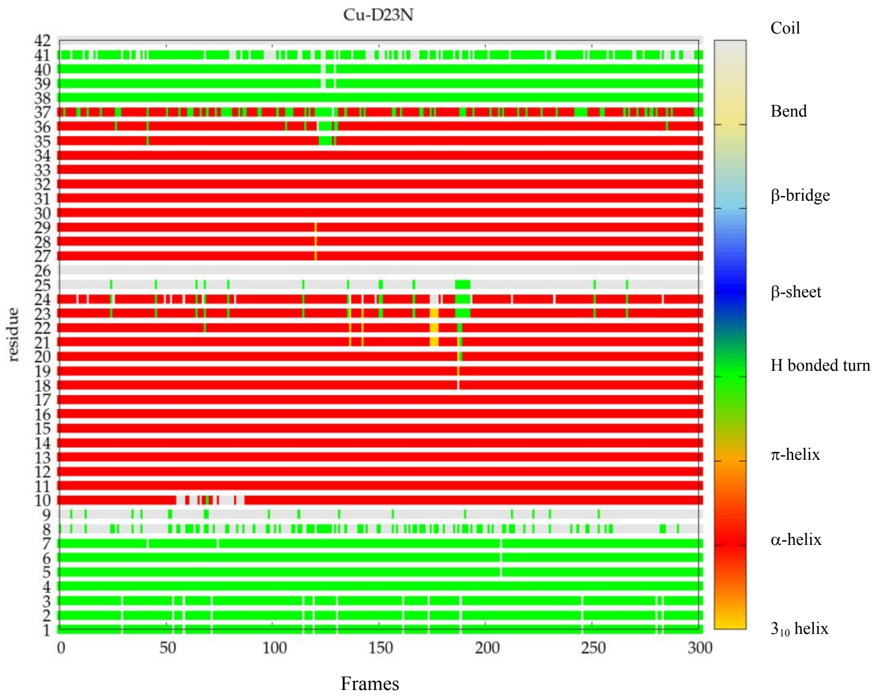

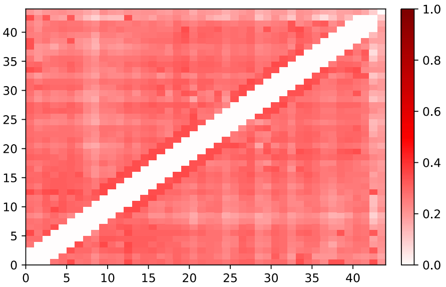

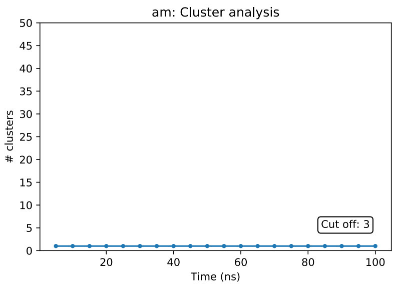

Figure S10: Converge analyses for Cu-D23N. A. Implicit solvent Free Energy and Radius of Gyration of the GaMD simulation, with the lowest energy structure. Timeline Analysis (B), RMSD all to all (C), PCA exploration (D), Contact Map (E) and Cluster Countering (F) of the MD simulation.

A

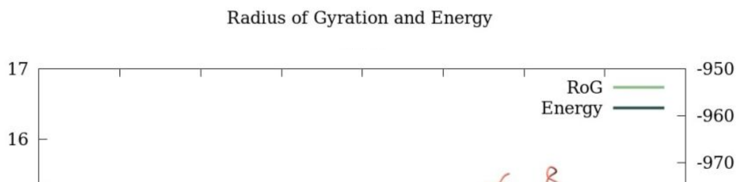

C

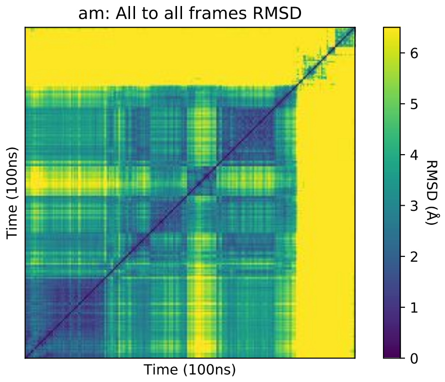

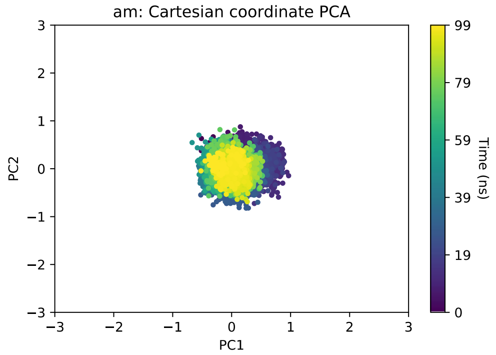

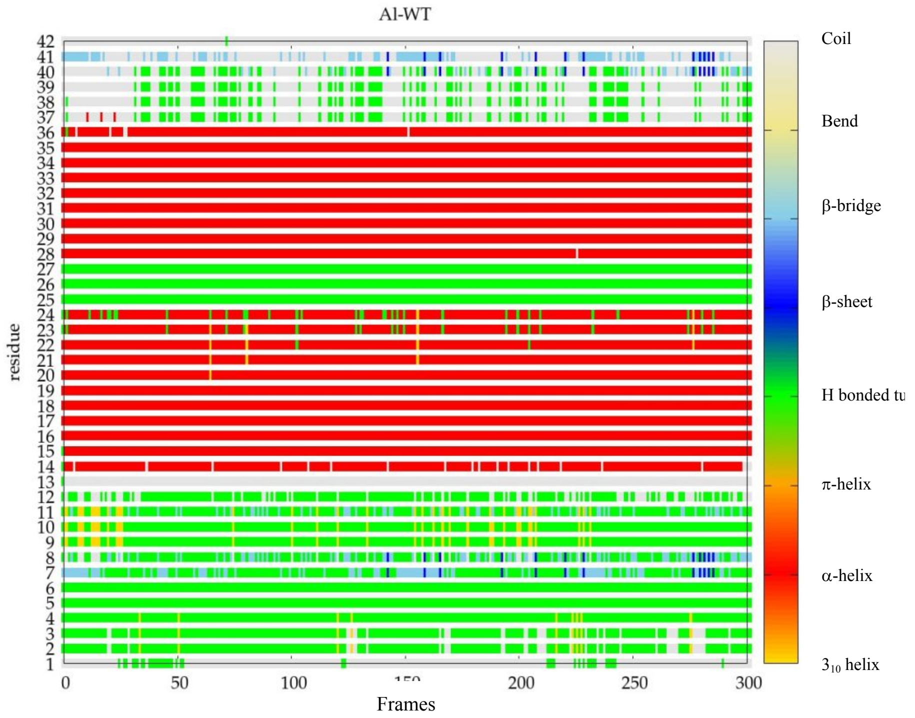

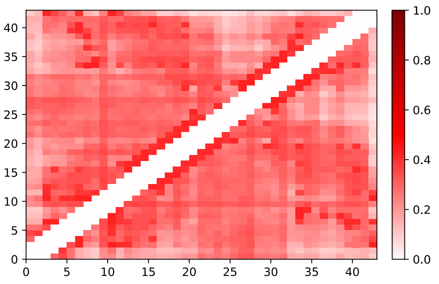

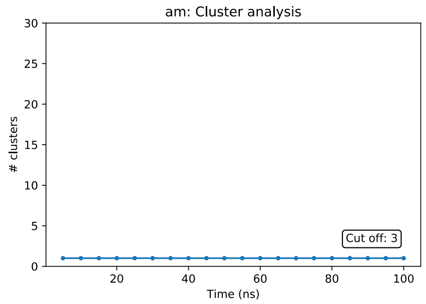

Figure S11: Converge analyses for Al-WT. A. Implicit solvent Free Energy and Radius of Gyration of the GaMD simulation, with the lowest energy structure. Timeline Analysis (B), RMSD all to all (C), PCA exploration (D), Contact Map (E) and Cluster Countering (F) of the MD simulation.

A

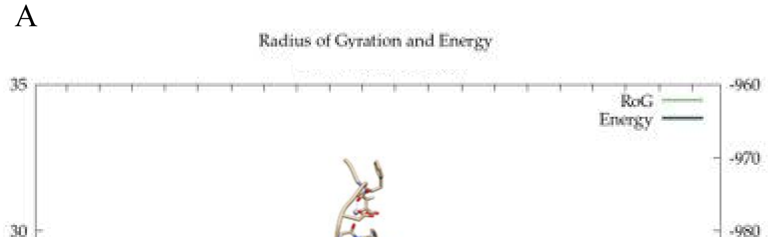

C

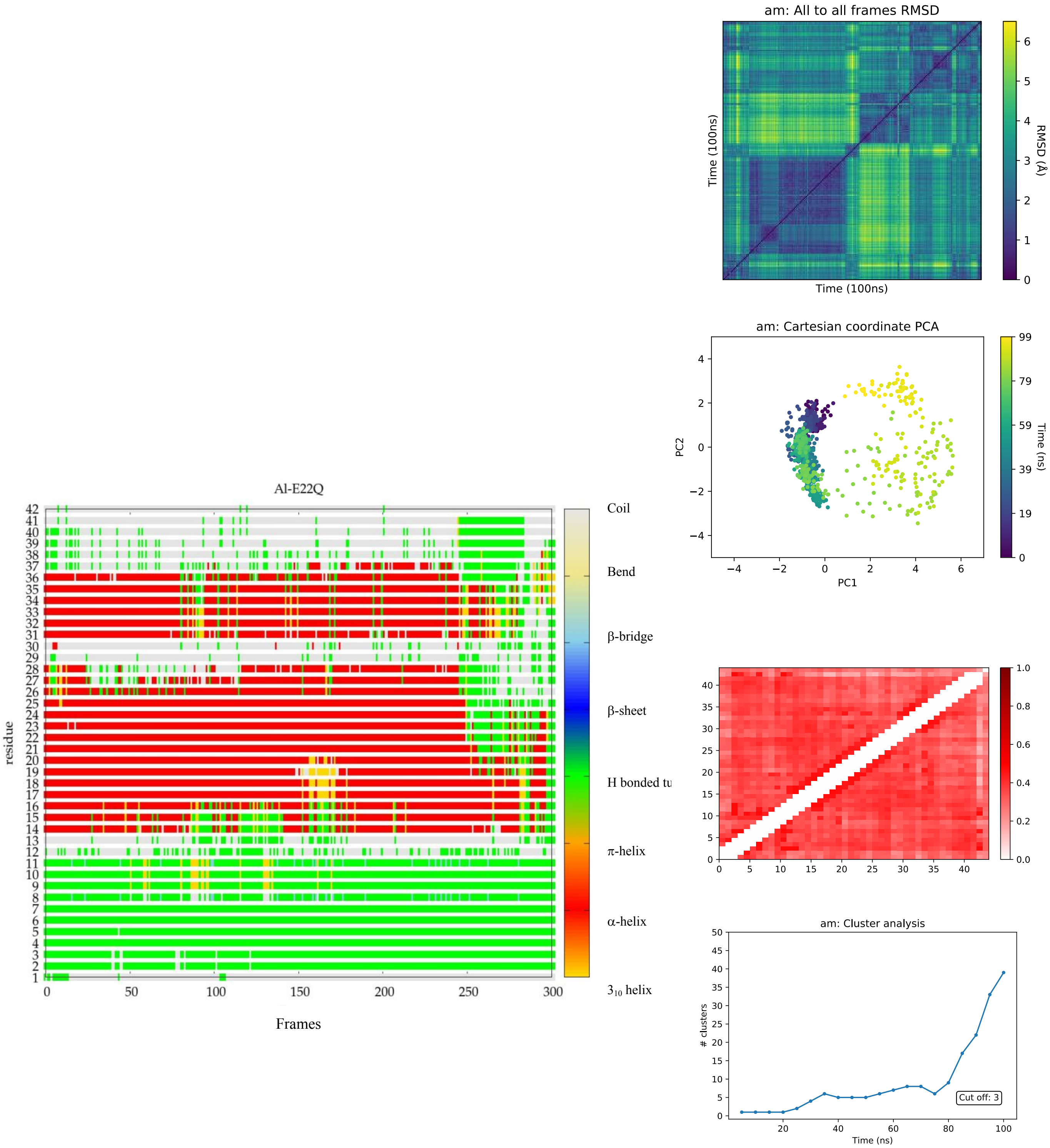

Figure S12: Converge analyses for Al-E22Q. A. Implicit solvent Free Energy and Radius of Gyration of the GaMD simulation, with the lowest energy structure. Timeline Analysis (B), RMSD all to all (C), PCA exploration (D), Contact Map (E) and Cluster Countering (F) of the MD simulation.

A

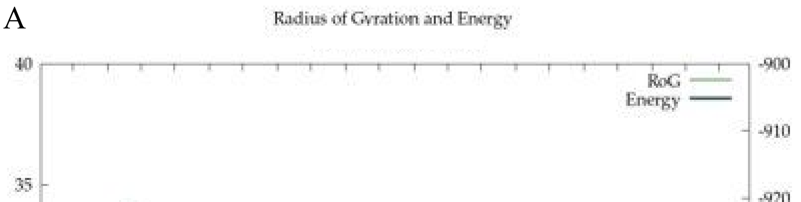

C

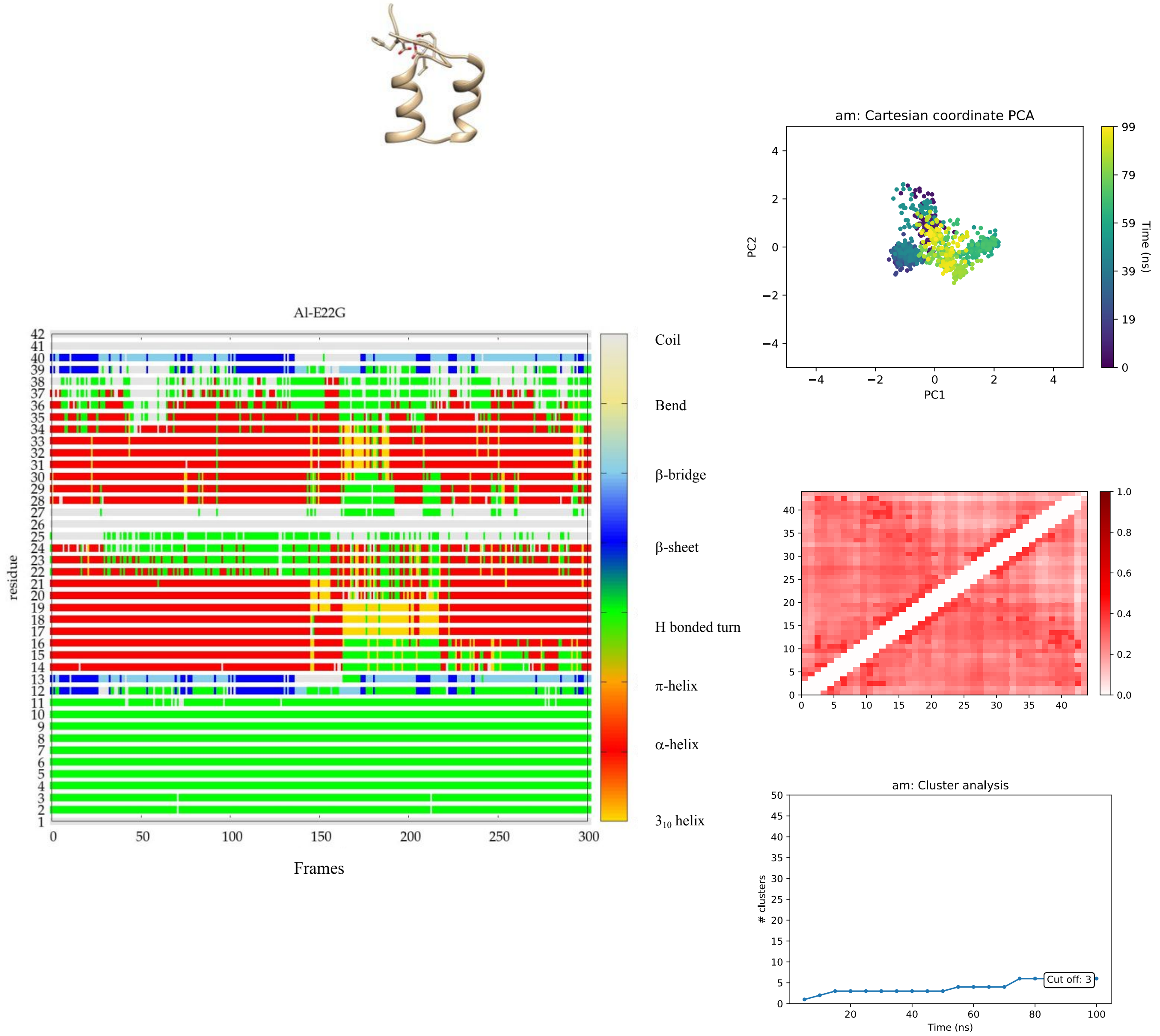

Figure S13: Converge analyses for Al-E22G. A. Implicit solvent Free Energy and Radius of Gyration of the GaMD simulation, with the lowest energy structure. Timeline Analysis (B), RMSD all to all (C), PCA exploration (D), Contact Map (E) and Cluster Countering (F) of the MD simulation.

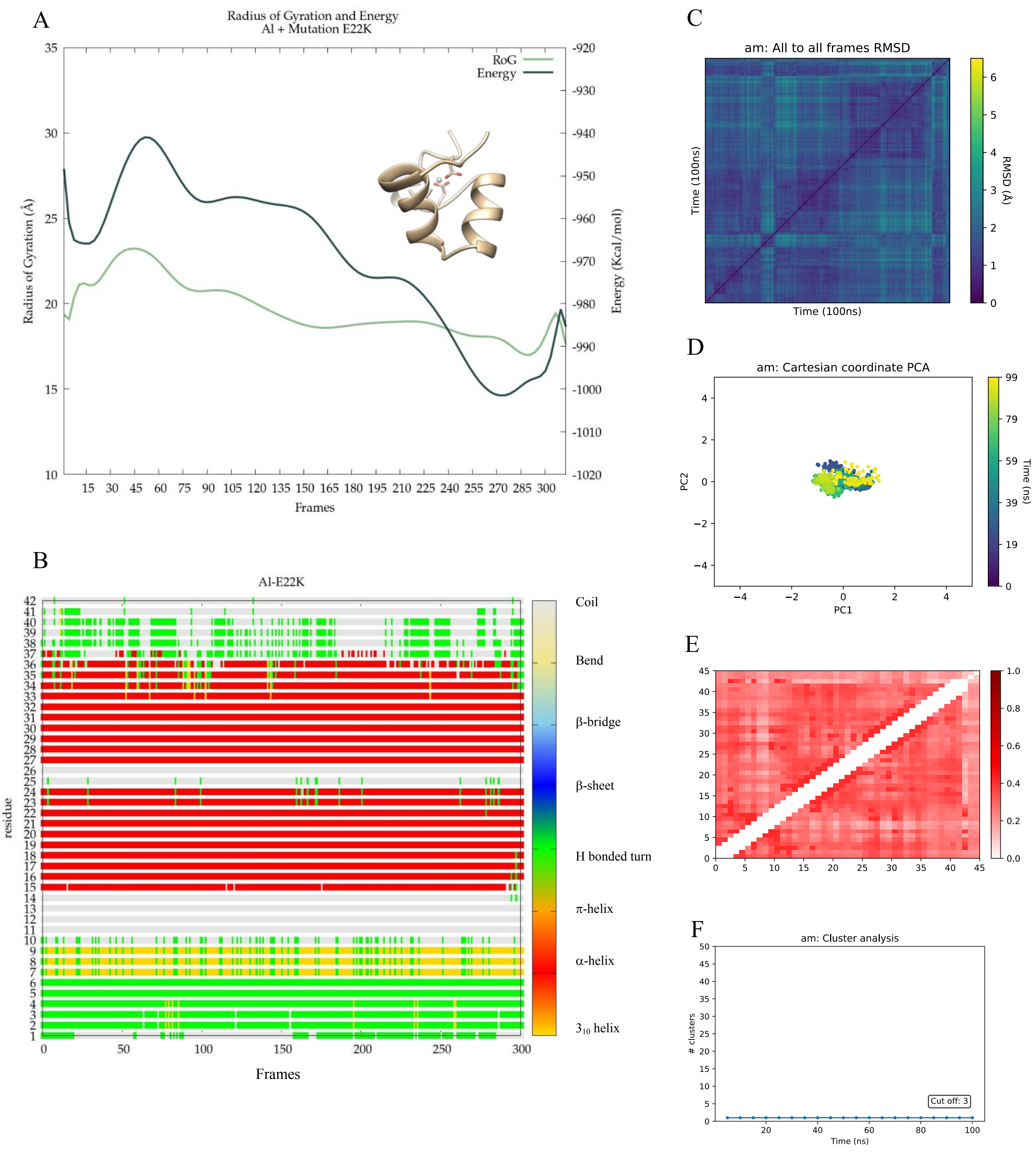

Figure S14: Converge analyses for Al-E22K. A. Implicit solvent Free Energy and Radius of Gyration of the GaMD simulation, with the lowest energy structure. Timeline Analysis (B), RMSD all to all (C), PCA exploration (D), Contact Map (E) and Cluster Countering (F) of the MD simulation.

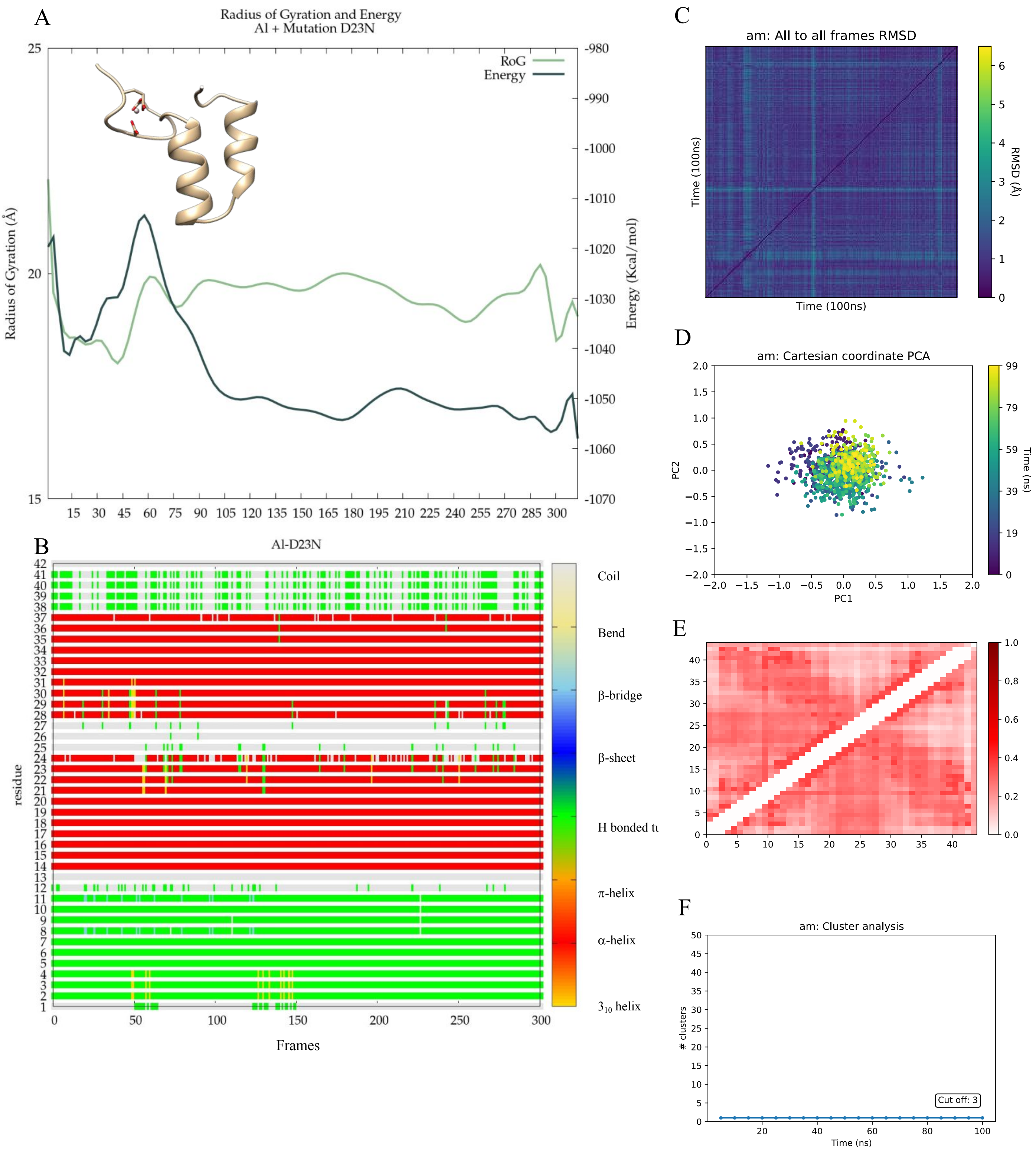

Figure S15: Converge analyses for Al-D23N. A. Implicit solvent Free Energy and Radius of Gyration of the GaMD simulation, with the lowest energy structure. Timeline Analysis (B), RMSD all to all (C), PCA exploration (D), Contact Map (E) and Cluster Countering (F) of the MD simulation.

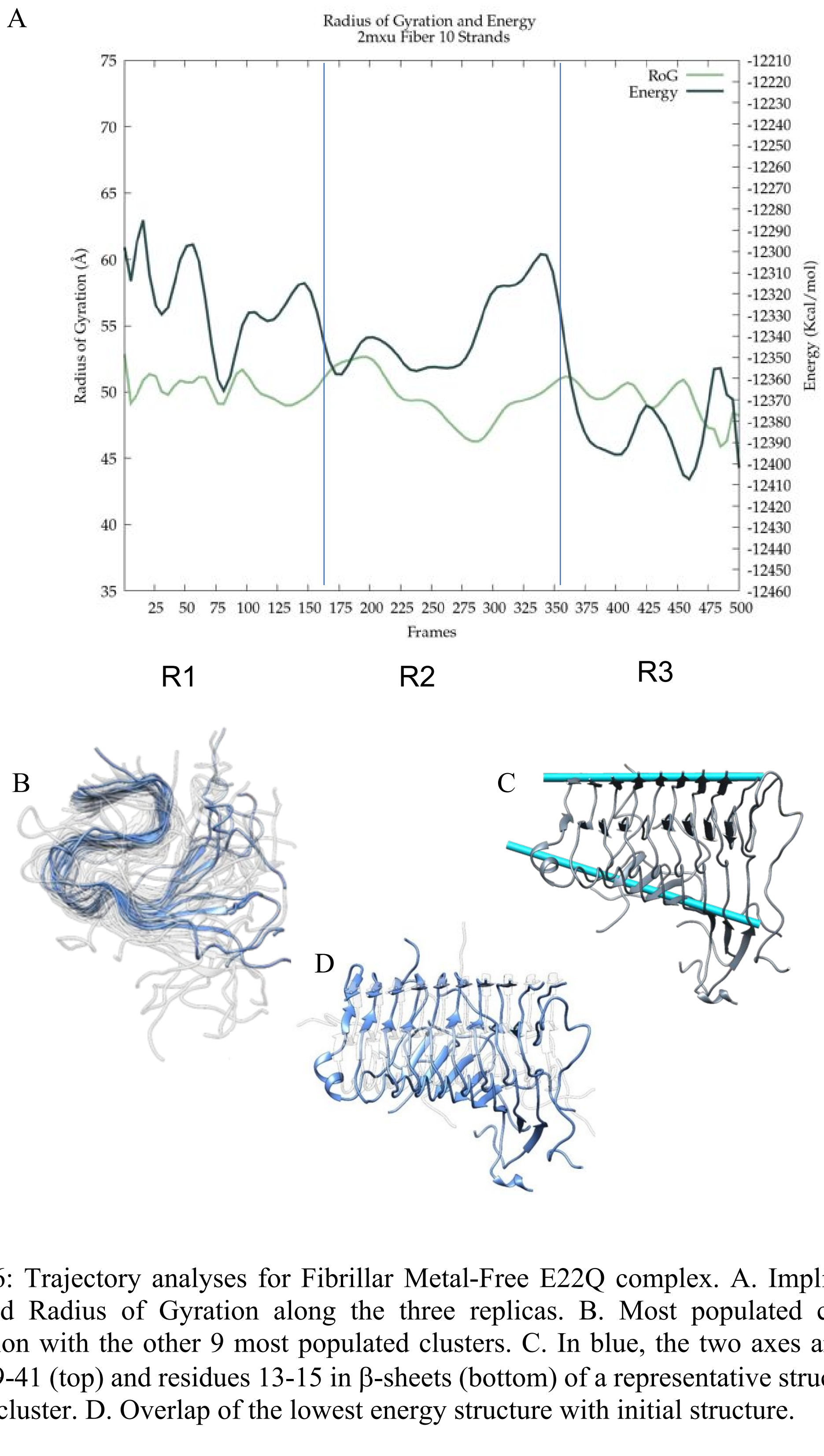

Figure S16: Trajectory analyses for Fibrillar Metal-Free E22Q complex. A. Implicit solvent Free Energy and Radius of Gyration along the three replicas. B. Most populated cluster (blue) in superposition with the other 9 most populated clusters. C. In blue, the two axes are comprised by residues 39-41 (top) and residues 13-15 in β-sheets (bottom) of a representative structure of the most populated cluster. D. Overlap of the lowest energy structure with initial structure.

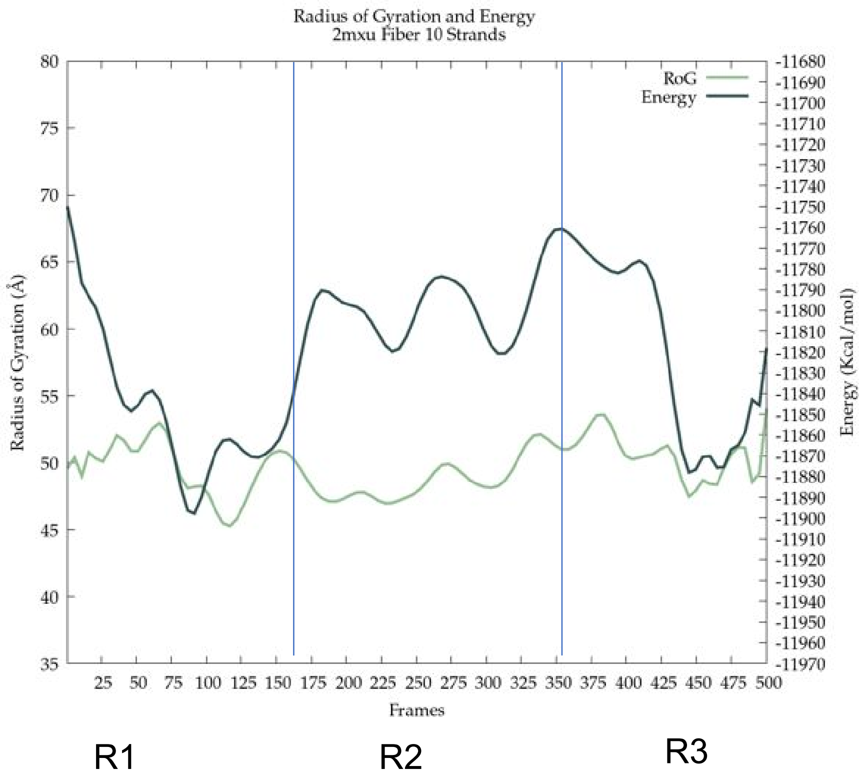

A

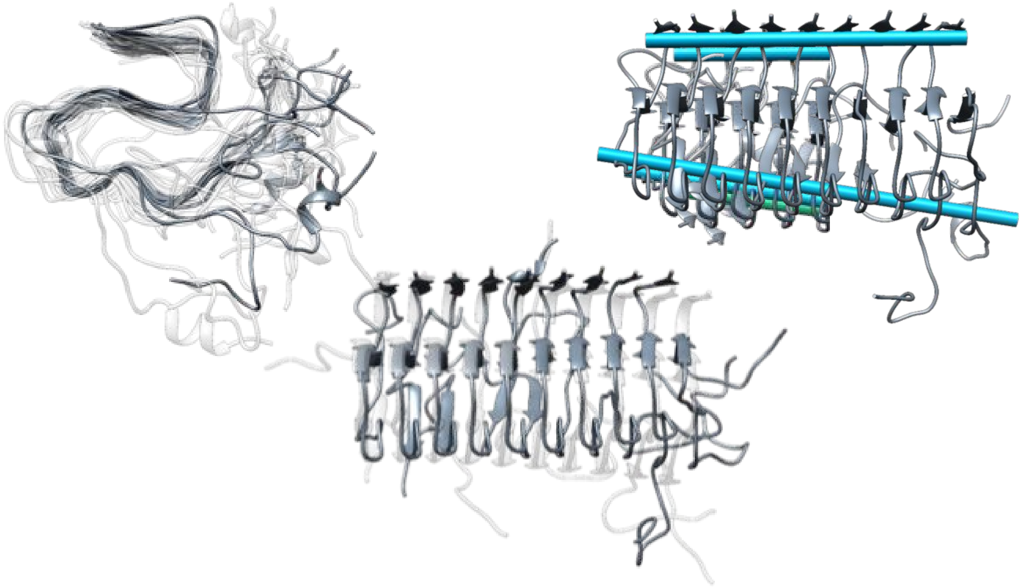

Figure S17: Trajectory analyses for Fibrillar Metal-Free E22G complex. A. Implicit solvent Free Energy and Radius of Gyration along the three replicas. B. Most populated cluster (blue) in superposition with the other 9 most populated clusters. C. In blue, the two axes are comprised by residues 39-41 (top) and residues 13-15 in β-sheets (bottom) of a representative structure of the most populated cluster. D. Overlap of the lowest energy structure w[illegible]

B C

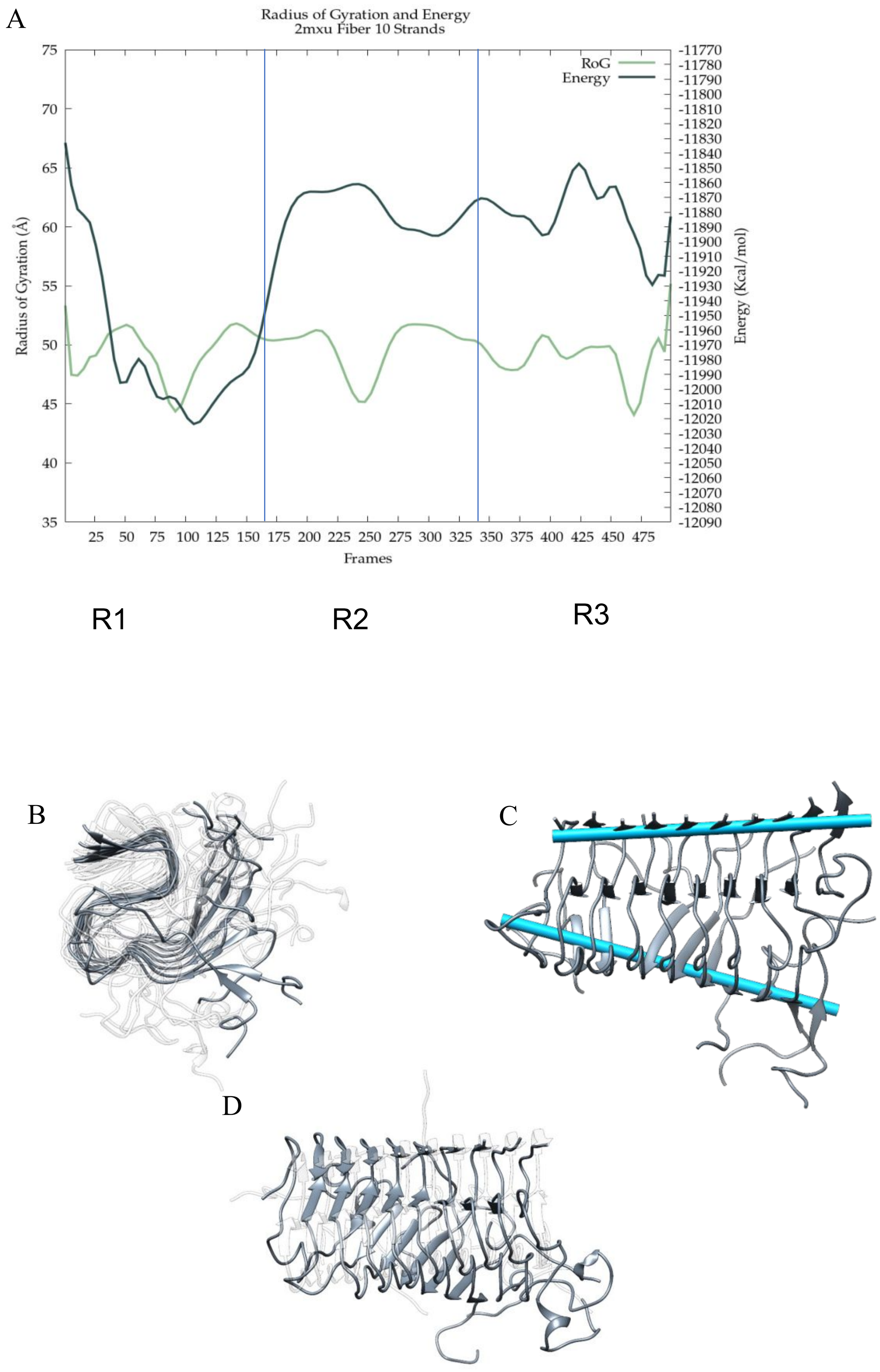

Figure S18: Trajectory analyses for Fibrillar Metal-Free E22K complex. A. Implicit solvent Free Energy and Radius of Gyration along the three replicas. B. Most populated cluster (blue) in superposition with the other 9 most populated clusters. C. In blue, the two axes are comprised by residues 39-41 (top) and residues 13-15 in β-sheets (bottom) of a representative structure of the most populated cluster. D. Overlap of the lowest energy structure with initial structure.

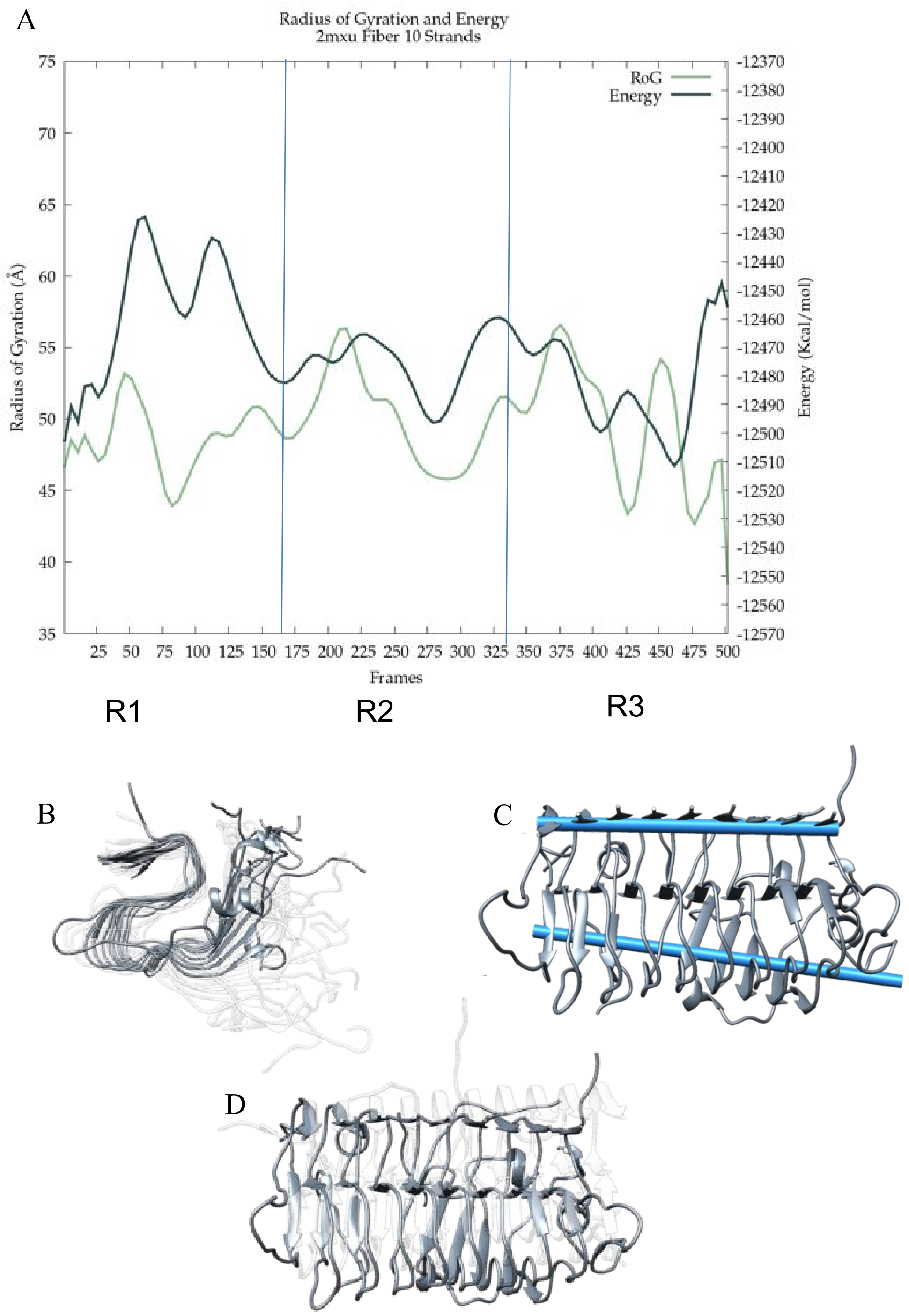

Figure S19: Trajectory analyses for Fibrillar Metal-Free D23N complex. A. Implicit solvent Free Energy and Radius of Gyration along the three replicas. B. Most populated cluster (blue) in superposition with the other 9 most populated clusters. C. In blue, the two axes are comprised by residues 39-41 (top) and residues 13-15 in β-sheets (bottom) of a representative structure of the most populated cluster. D. Overlap of the lowest energy structure with initial structure.

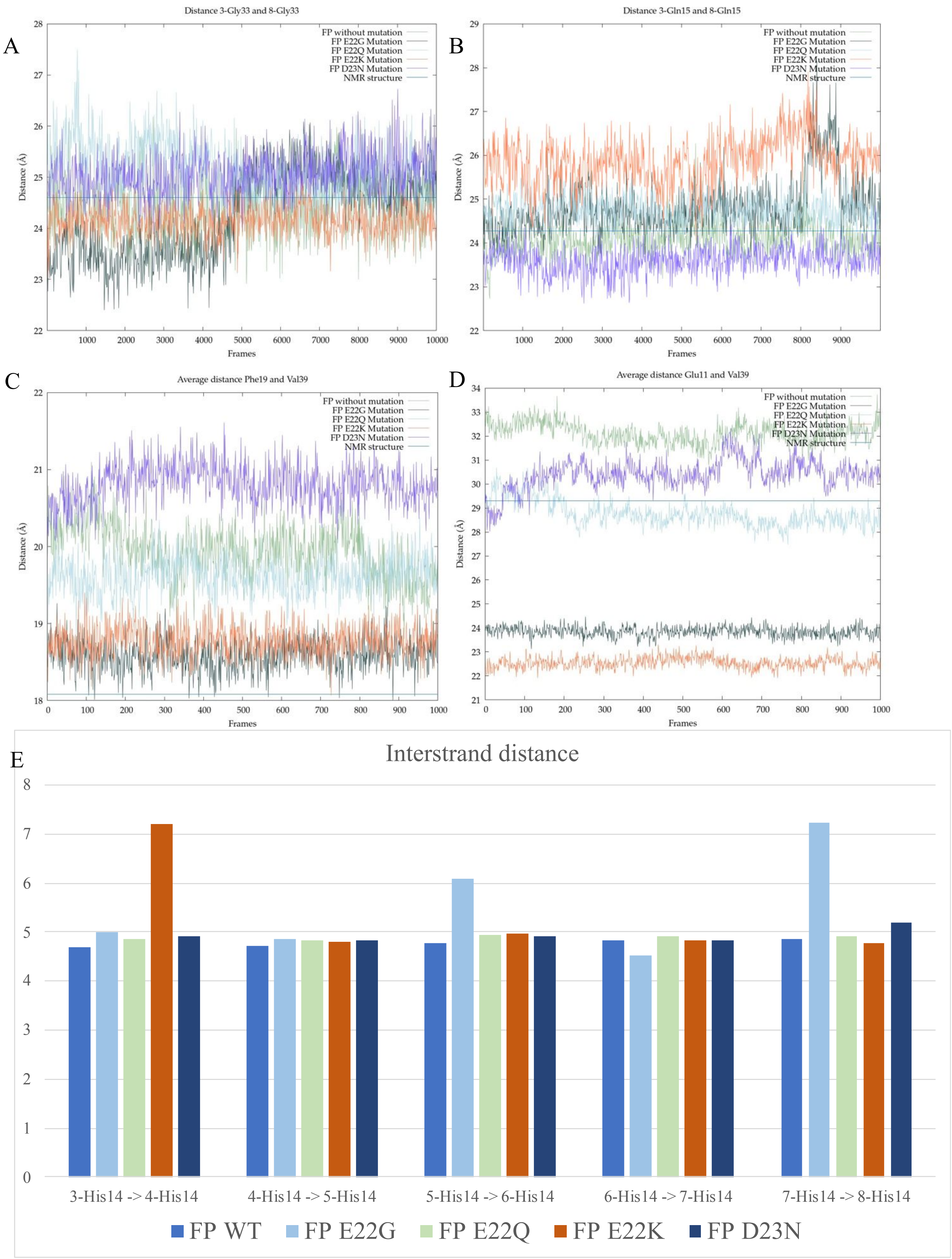

Figure S20: Metal-free complexes' measures of A. 3-Gy33 and 8-Gly33(**M_{HC}**), B. 3-Gln15 8-Gln15 measure (**M_{HE}**), C. Phe19-Val39 (**M_{VC}**), D. Glu11-Val39 measure (**M_{VE}**) for WT (light green), E22Q (light blue), E22G (dark green), E22K (orange) and D23N (purple) variants. E. Measure of interstrand distances (**M_{IS}**).

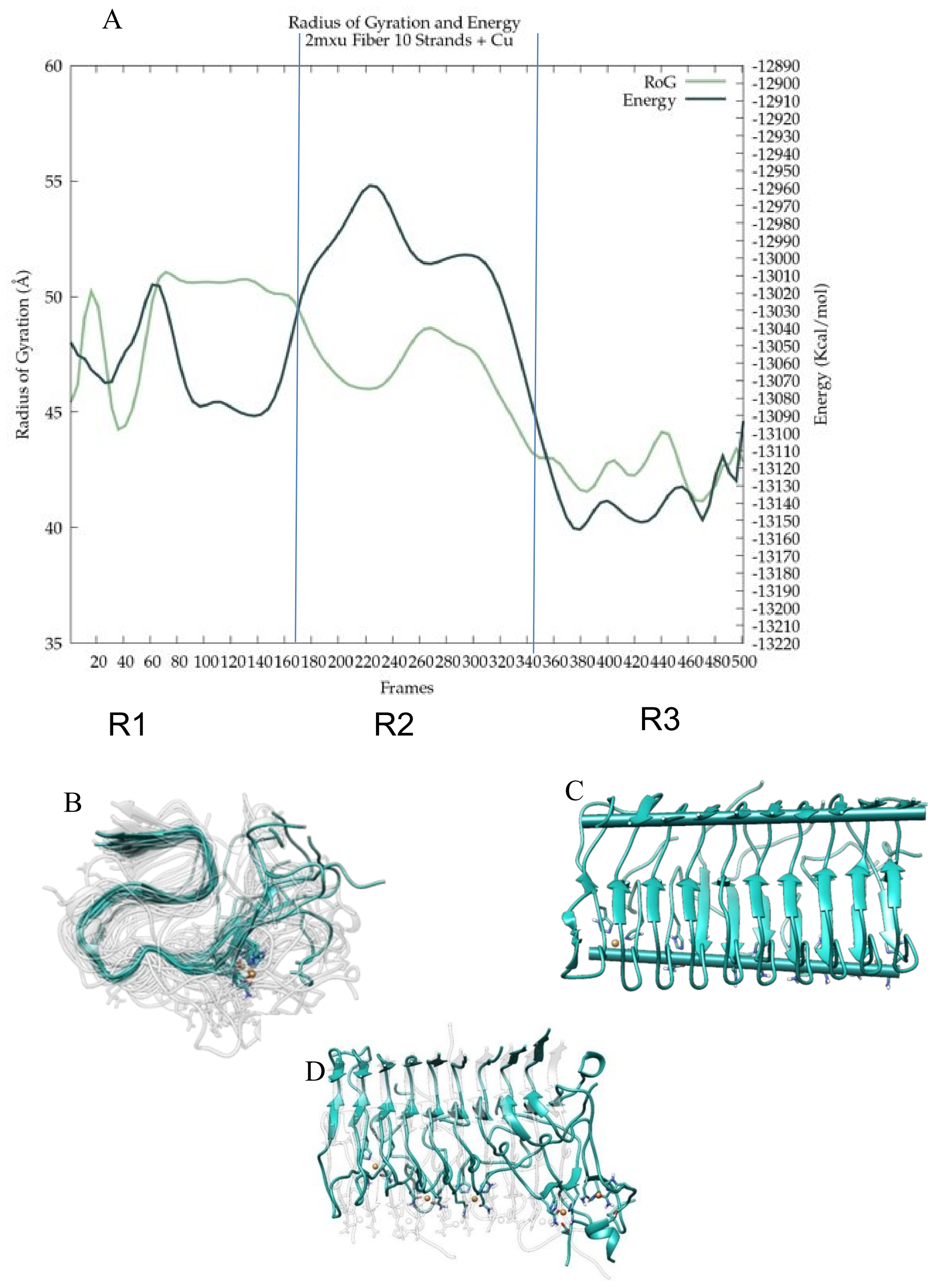

Figure S21: Trajectory analyses for Fibrillar Cu-E22Q complex. A. Implicit solvent Free Energy and Radius of Gyration along the three replicas. B. Most populated cluster (blue) in superposition with the other 9 most populated clusters. C. In blue, the two axes are comprised by residues 39-41 (top) and residues 13-15 in β-sheets (bottom) of a representative structure of the most populated cluster. D. Overlap of the lowest energy structure with initial structure.

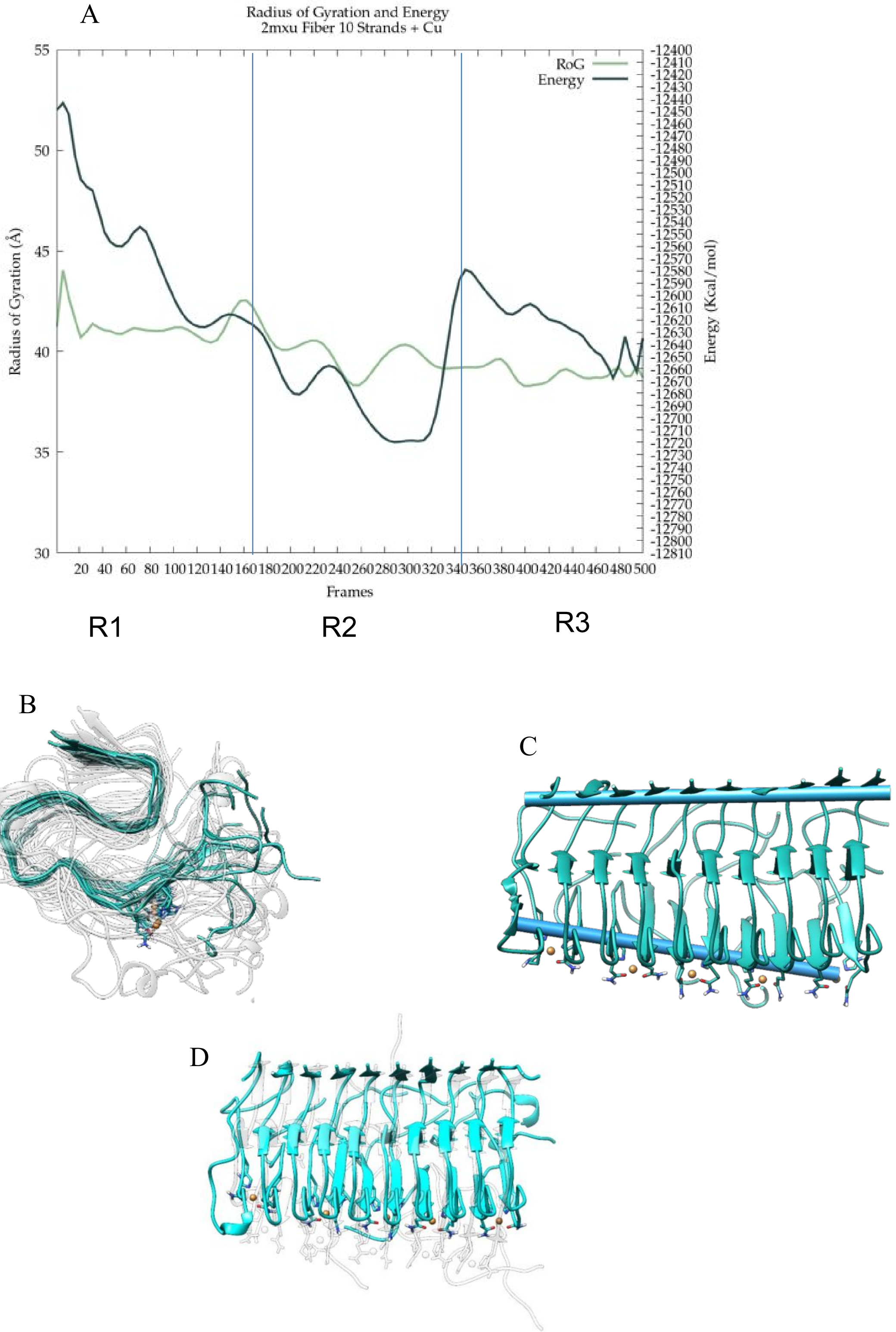

Figure S22: Trajectory analyses for Fibrillar Cu-E22G complex. A. Implicit solvent Free Energy and Radius of Gyration along the three replicas. B. Most populated cluster (blue) in superposition with the other 9 most populated clusters. C. In blue, the two axes are comprised by residues 39-41 (top) and residues 13-15 in β-sheets (bottom) of a representative structure of the most populated cluster. D. Overlap of the lowest energy structure with initial structure.

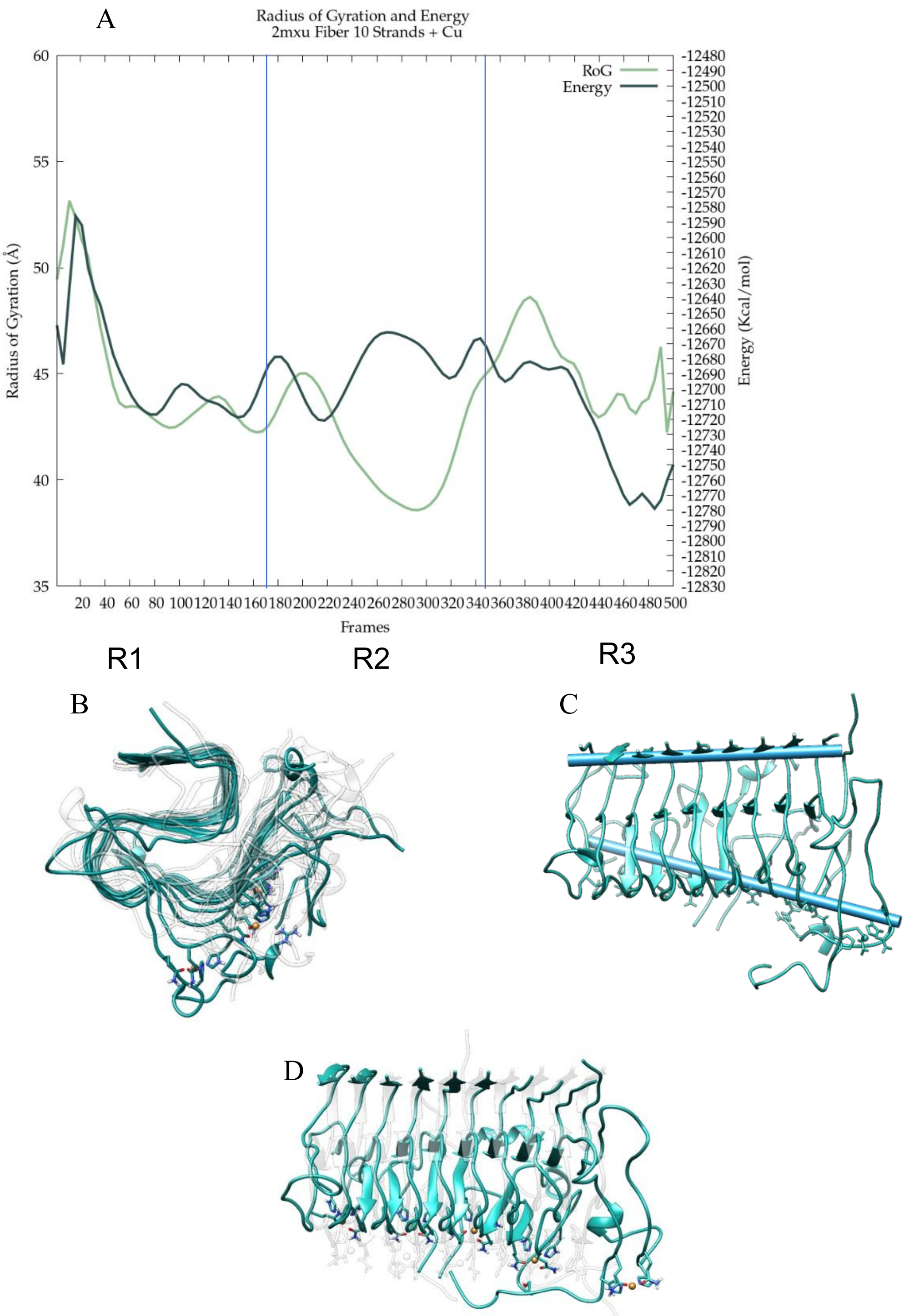

Figure S23: Trajectory analyses for Fibrillar Cu-E22K complex. A. Implicit solvent Free Energy and Radius of Gyration along the three replicas. B. Most populated cluster (blue) in superposition with the other 9 most populated clusters. C. In blue, the two axes are comprised by residues 39-41 (top) and residues 13-15 in β-sheets (bottom) of a representative structure of the most populated cluster. D. Overlap of the lowest energy structure with initial structure.

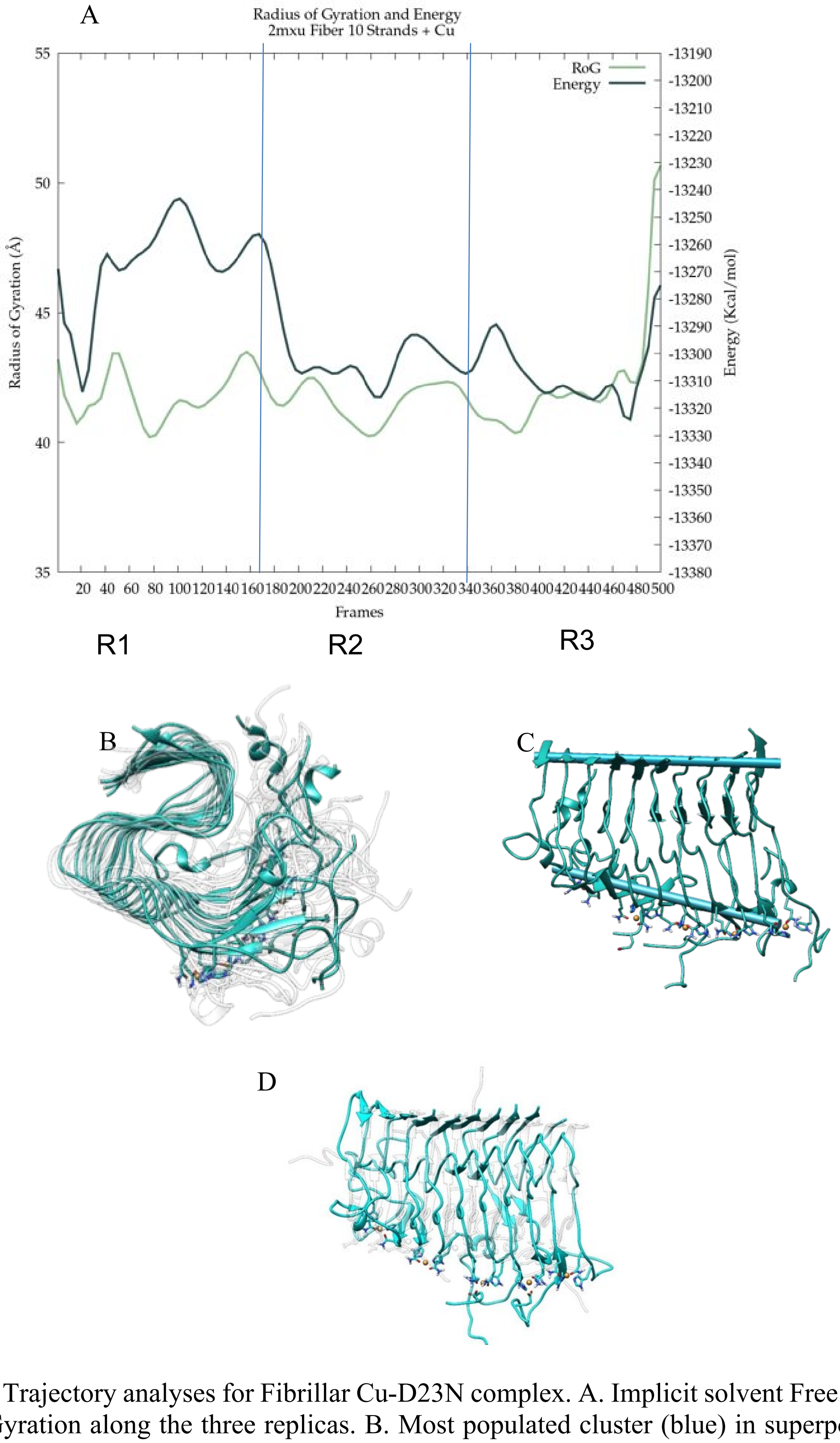

Figure S24: Trajectory analyses for Fibrillar Cu-D23N complex. A. Implicit solvent Free Energy and Radius of Gyration along the three replicas. B. Most populated cluster (blue) in superposition with the other 9 most populated clusters. C. In blue, the two axes are comprised by residues 39-41 (top) and residues 13-15 in β-sheets (bottom) of a representative structure of the most populated cluster. D. Overlap of the lowest energy structure with initial structure.

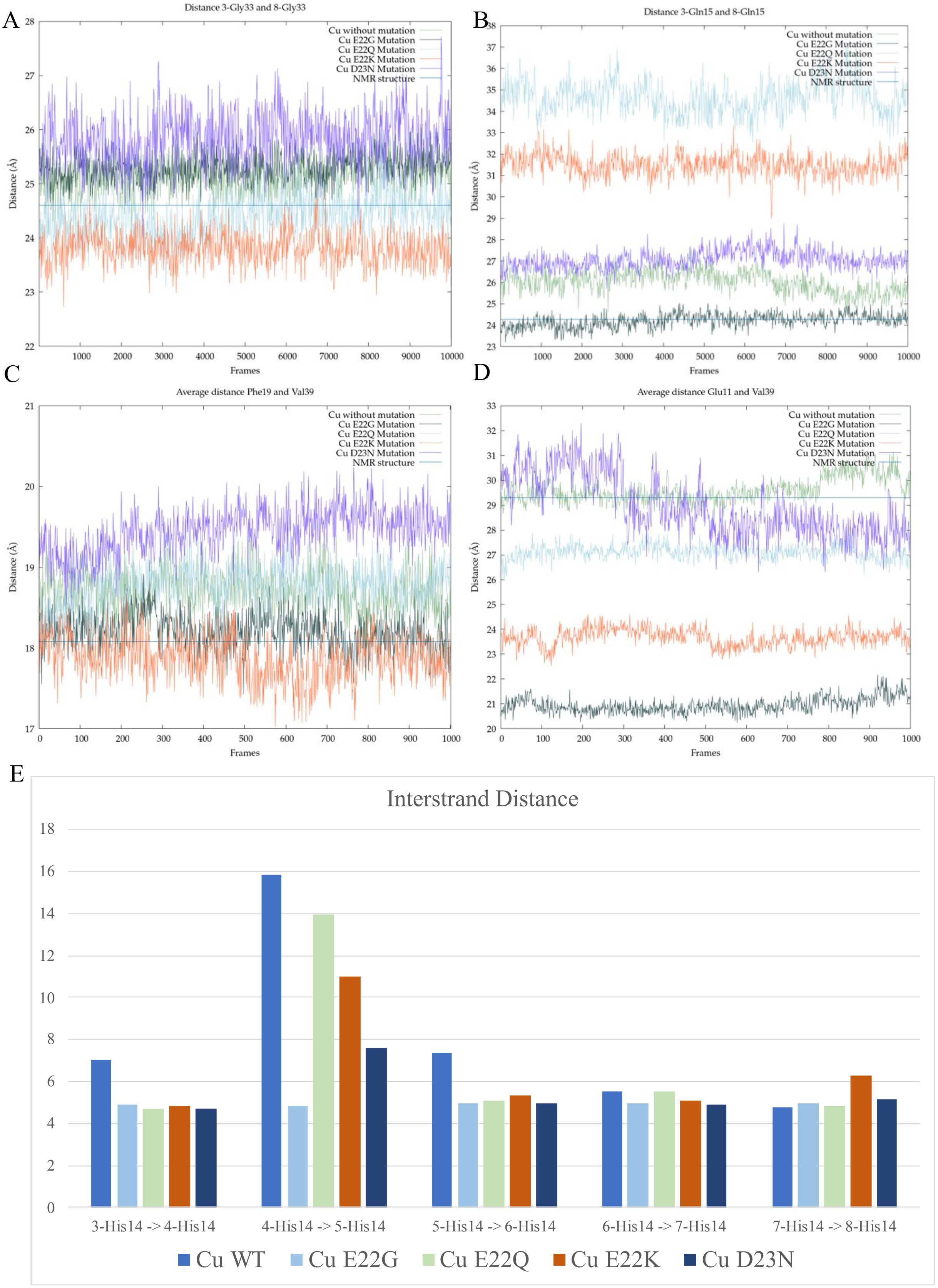

Figure S25: Cu(II) bound complexes' measures of A. 3-Gy33 and 8-Gly33(**M$_{HC}$**), B. 3-Gln15 8-Gln15 measure (**M$_{HE}$**), C. Phe19-Val39 (**M$_{VC}$**), D. Glu11-Val39 measure (**M$_{VE}$**) for WT (light green), E22Q (light blue), E22G (dark green), E22K (orange) and D23N (purple) variants. E. Measure of interstrand distances (**M$_{IS}$**).

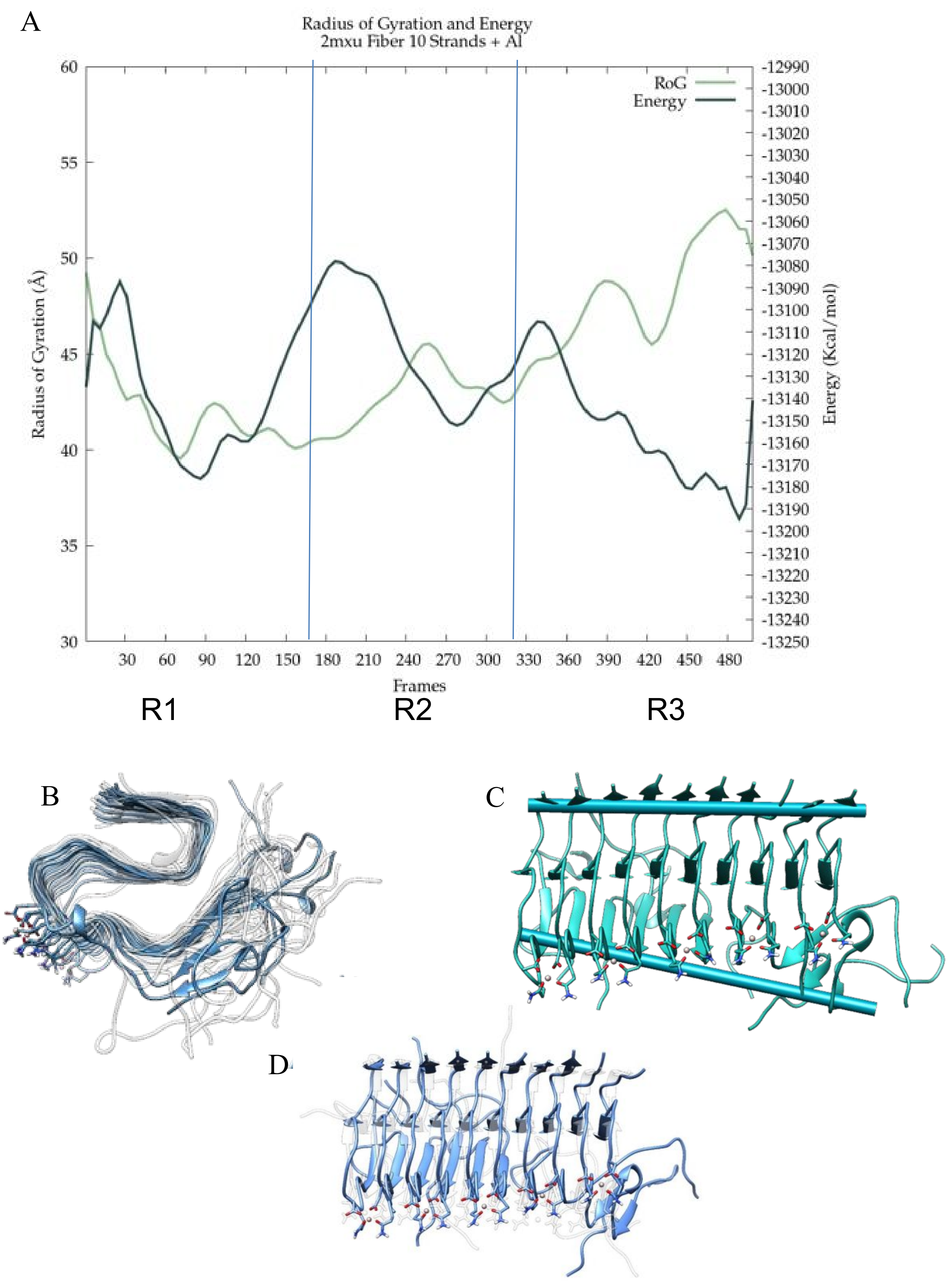

Figure S26: Trajectory analyses for Al-E22Q complex. A. Implicit solvent Free Energy and Radius of Gyration along the three replicas. B. Most populated cluster (blue) in superposition with the other 9 most populated clusters. C. In blue, the two axes are comprised by residues 39-41 (top) and residues 13-15 in β-sheets (bottom) of a representative structure of the most populated cluster. D. Overlap of the lowest energy structure with initial structure.

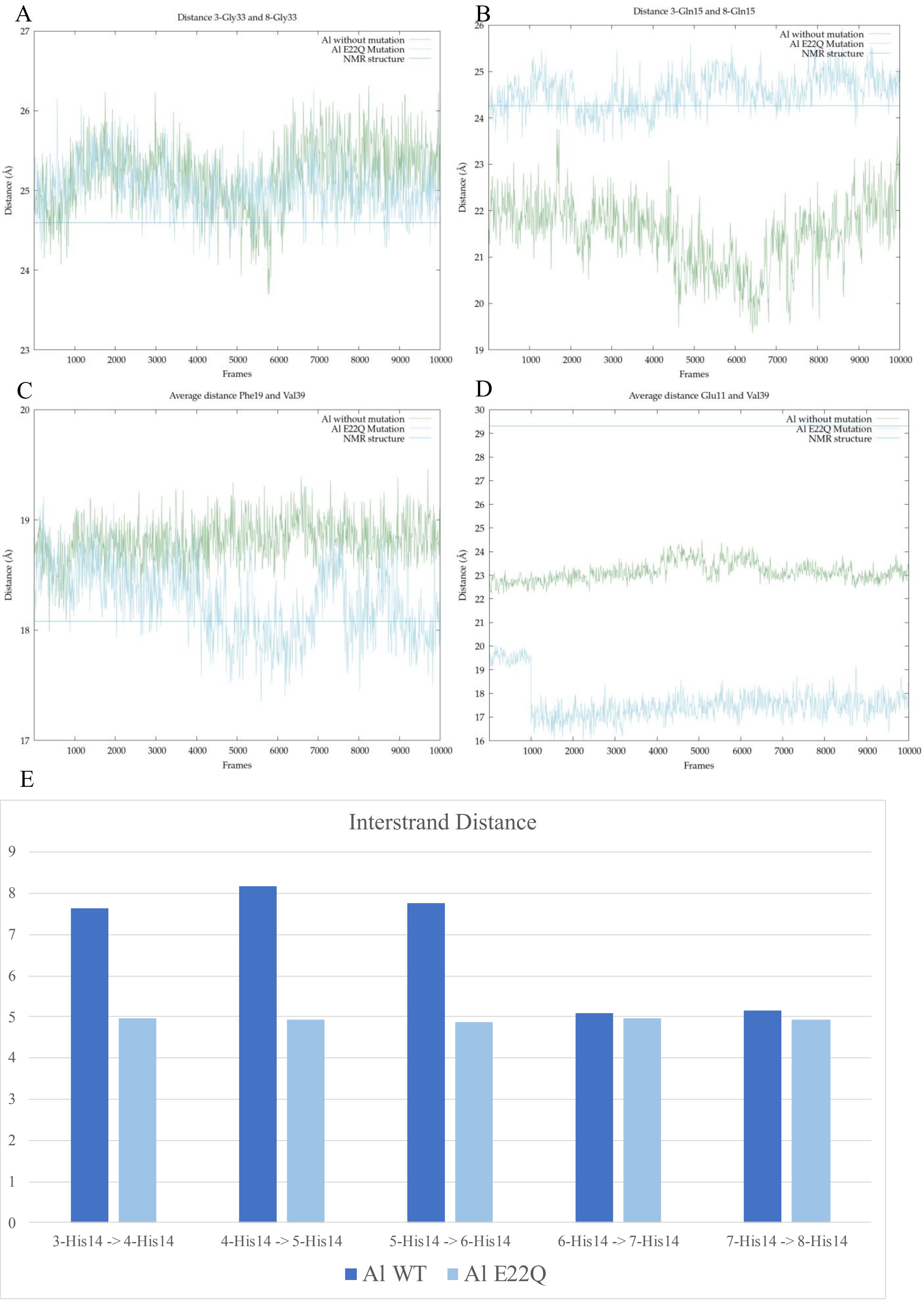

Figure S27: Al-bound complexes' measures of A. 3-Gy33 and 8-Gly33($\mathbf{M}_{\mathbf{HC}}$), B. 3-Gln15 8-Gln15 measure ($\mathbf{M}_{\mathbf{HE}}$), C. Phe19-Val39 ($\mathbf{M}_{\mathbf{VC}}$), D. Glu11-Val39 measure ($\mathbf{M}_{\mathbf{VE}}$) for WT (light green), E22Q (light blue), E22G (dark green), E22K (orange) and D23N (purple) variants. E. Measure of interstrand distances ($\mathbf{M}_{\mathbf{IS}}$).
